# Space-based observation of global increase in urban methane emissions from 2019–2023

**DOI:** 10.1073/pnas.2504211123

**Published:** 2026-04-13

**Authors:** Erica Whiting, Genevieve Plant, Eric A. Kort, Ilse Aben, Kira J. Biener, Gijs Leguijt, Joannes D. Maasakkers

**Affiliations:** ^a^Climate and Space Sciences and Engineering, University of Michigan, Ann Arbor, MI 48109; ^b^Atmospheric Chemistry Department, Max Planck Institute for Chemistry, Mainz 55128, Germany; ^c^Netherlands Institute for Space Research, Leiden 2333 CA, The Netherlands; ^d^Earth and Planetary Sciences, Yale University, New Haven, CT 06511; ^e^Department of Climate, Air and Sustainability, Netherlands Organisation for Applied Scientific Research, Utrecht 3584 CB, The Netherlands

**Keywords:** methane, urban, remote-sensing, C40, TROPOMI

## Abstract

Cities around the world have united to form coalitions, like the C40 network, in pursuit of ambitious climate goals. These efforts often include reducing methane emissions. However, sources and magnitudes of urban methane emissions are not well known, and there is not currently a method to evaluate implementation of mitigation measures. Here, we fill this observational gap with a tracer–tracer approach using space-based observations of methane and carbon monoxide from the TROPOspheric Monitoring Instrument satellite instrument. We measure methane emissions of 92 global cities, including their broader metropolitan area, and find aggregate emissions of 31.2 Tg CH_4_/y (95%CI: 22.3, 40.4 Tg CH_4_/y) in 2023, equivalent to ∼10% of the global anthropogenic methane budget. We track emissions for 72 of these cities (51 C40 cities and 21 non-C40 cities) from 2019–2023. Methane emissions from these cities weakly declined in 2020 followed by steady growth, with a 2.3 Tg aggregate increase over 4 y. This growth contributes minimally to the recent atmospheric methane surge. While C40 cities have largely pledged 34% reductions by 2030, we observe significant growth from 2020 to 2023 (10%, 95%CI: 2%, 17%), similar to growth observed in non-C40 cities (12%, 95%CI: −1.5%, 25%). Inventories fail to capture observed growth, suggesting urban emissions are not well characterized, and mitigation approaches may not be optimally designed. For the C40 network to achieve its methane target (34% by 2030), steep, rapid reductions will be needed. Emission reductions of this magnitude would be detectable with the space-based approach used in this work.

## Significance

Cities are large emitters of methane, a potent greenhouse gas and precursor for tropospheric ozone. We use space-based observations to study nearly 100 cities with a total population of 1.18 billion people, and find emissions from these cities to have substantial global impact, equal to 3.75 times the contribution of recently reported oil and gas “Ultra-Emitters.” Across most cities we observe increasing urban CH_4_ emissions regardless of participation in the C40 climate pledge. Our findings emphasize the large and growing impact of urban CH_4_ emissions, suggesting that successful urban mitigation approaches would have large benefit. We show here that satellite-based observations can provide guidance to whether emissions estimates and policies are accurate and effective.

Cities are home to more than half of the global population (1) and nearly 70% of world’s anthropogenic greenhouse gas emissions (2), many of which have the motivation and political power to implement climate policies. Methane (CH_4_) has been identified as a key target in reducing anthropogenic greenhouse gas emissions (3), as it is a strategic lever in limiting warming on decadal timescales due to its short lifetime (4). Urban areas contribute substantially to the total methane budget and host diverse combinations of sectors including landfills, wastewater handling, and fossil fuel activities, from distribution through end use (5). Reducing urban CH_4_ emissions from these sources could have a substantial impact on the global CH_4_ budget.

Many cities have united behind pledges and coalitions, such as the C40 city network, to reduce greenhouse gas emissions, including CH_4_. C40, first founded in 2005, is a coalition of now 97 member cities who have made pledges to cut “their fair share of greenhouse gas emissions” in half by 2030 and reach emissions neutrality by 2050 (6). The C40-supported UN Race to Zero, as part of its criteria for leadership practices, calls for a reduction in CH_4_ emissions of at least 34% by 2030 (7). C40 strongly encourages even more stringent 50% reductions by 2030 (8) and hosts targeted initiatives like the C40 Pathway Towards Zero Waste which focuses on reducing CH_4_ emissions with a waste-sector specific approach (9, 10). However, to ensure these ambitious pledges translate into effective action, cities need reliable feedback on their mitigation efforts. Previous studies of urban CH_4_ have indicated that bottom–up inventories, which can inform mitigation planning, often underestimate urban CH_4_ emissions, suggesting that the sources and magnitudes of these emissions are not well understood (11–35). Additionally, reported CH_4_ emissions vary largely in global bottom–up inventory versions. Without atmospheric monitoring, it is challenging to evaluate whether mitigation measures are effectively targeted and implemented.

Urban CH_4_ emissions have typically been evaluated using ground-based and airborne instruments, which often require atmospheric transport models to link observations to emissions. Until recently, studies have focused primarily on cities in Western Europe and North America, often observing emissions over limited time periods (11–26, 29–32, 35–41). Due to the finite nature of these campaigns, we are unable to examine emissions over seasonal and interannual timescales or at a global scale. Additionally, cities have unique infrastructure, waste management, and natural gas distribution systems and studies confined to North America and Europe may not comprehensively represent total urban emissions. Thus, there is a gap in understanding how cities around the world contribute to the global annual CH_4_ budget. In addition to lacking understanding of baseline urban CH_4_ emissions, many cities around the world are making pledges to reduce CH_4_ emissions without observational systems for monitoring urban-scale emissions. Space-based approaches offer a method to quantify and monitor CH_4_ emissions in a consistent, on-going manner with global coverage (42). Recent studies use satellites combined with auxiliary meteorological inputs (e.g. transport model) to quantify urban CH_4_ emissions (27–31). These methods are often limited in time or by region due to method complexity and utility, hindering the holistic understanding needed to support effective urban mitigation strategies.

Here, we build upon the Plant et al. (25) application of a satellite-based tracer–tracer analysis between CH_4_ and carbon monoxide (CO) over US cities to calculate urban CH_4_ emissions. Tracer–tracer analysis leverages the concentration ratio between two trace gases to infer the emissions of one trace gas using the better-known emissions of the other (11, 19, 21, 25, 33, 34, 43–45). Methane and CO are not coemitted at the surface from the same sources. They are, however, both emitted in the urban domain. If the urban emissions of these gases dominate the enhancement in the column retrievals, a characteristic relationship emerges at the city scale (25). The gases in this relationship are impacted by the same meteorology, allowing for use of tracer–tracer analysis when the city is viewed as an integrated source (25). The relationship between the two observed quantities is used to calculate an enhancement ratio. Because the sources of CO are relatively well quantified and reported compared to CH_4_, the enhancement ratio is used to relate the city’s CH_4_ emissions to the relatively well-known CO emissions. Plant et al. (25) evaluated the approach and found agreement with results from simulations and airborne measurements, as well as finding urban emissions to be underestimated in inventories for the limited cities studied. This data-driven method offers a low-latency solution that can be applied generally to urban areas across the world.

Building upon these findings, we employ this low-latency, data-driven approach using the TROPOspheric Monitoring Instrument (TROPOMI) on board the Sentinel-5 Precursor satellite to calculate daily CH_4_:CO enhancement ratios, which are robust observables independent of modeling (46, 47). In this work, we quantify the CH_4_ emissions of 92 cities across the world. For our purpose, we consider cities to include the large metropolitan area with the city at its core (for more details see *SI Appendix*). Additionally, we track emissions over 5 y (2019–2023) of 51 cities that are a part of the C40 climate pledge and of 21 non-C40 cities. This work demonstrates the use of satellite measurements as an ongoing monitoring tool that can provide guidance to emissions reduction strategies.

## Results

**Fig. 1.**
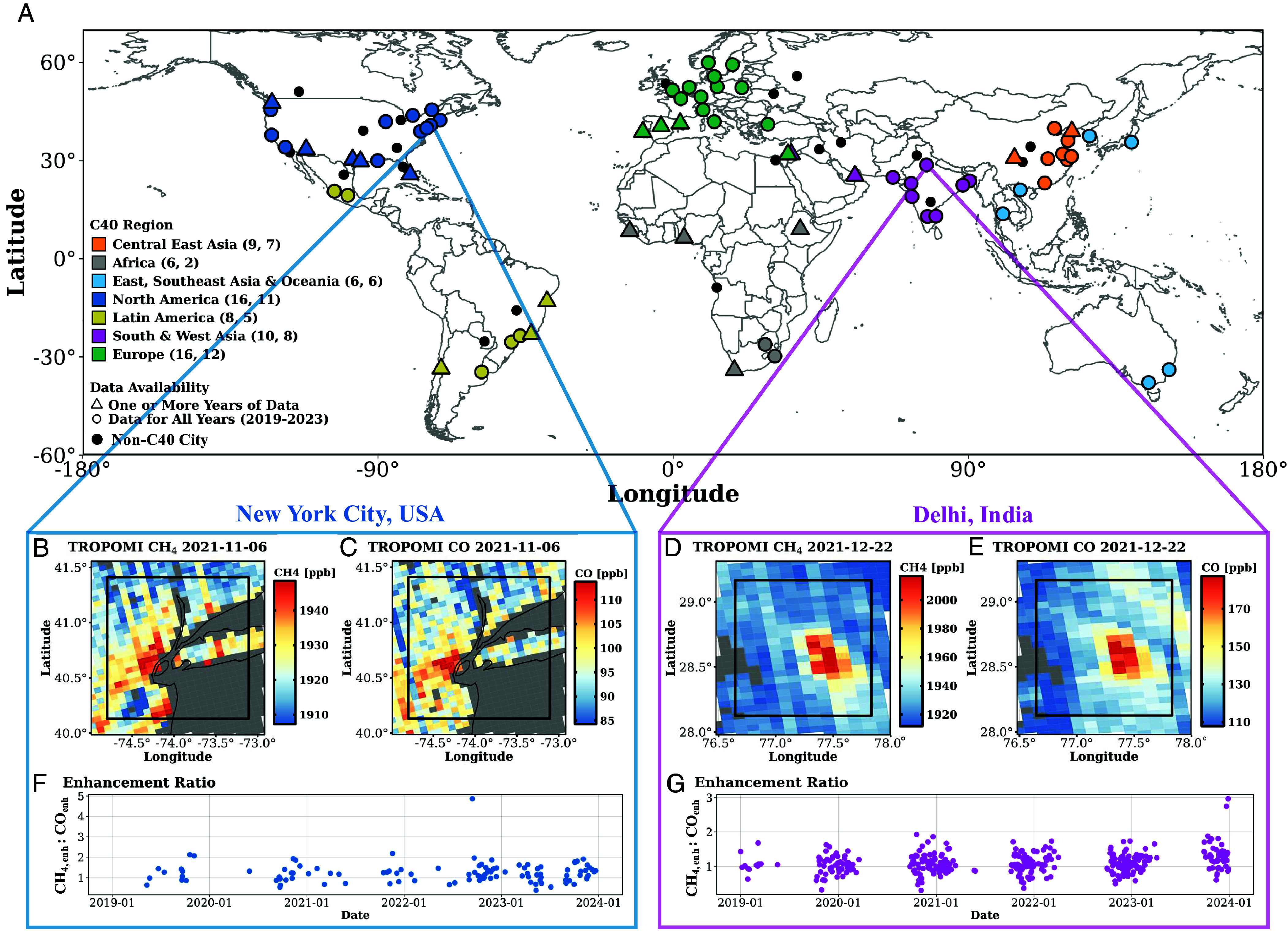
Global map of city locations and examples showing domain selection, TROPOMI CH_4_ and CO overpasses, and time series of the enhancement ratio. (*A*) shows the city locations for C40 and non-C40 members. Non-C40 cities, shown by a small black point, have data for every year (2019–2023). C40 cities can be shown by a triangle for having at least one year of data after filtering, or by a circle for having data for every year (2019–2023), colored by C40-designated region. The number of cities in each region is shown first in parentheses, followed by the number of cities with data for all years in that region. We have observations for at least one year of data for 71 C40 cities. We have observations across all years for 51 of these C40 cities along with 21 non-C40 cities. (*B*–*E*) show the dry column mixing ratios from TROPOMI CH_4_ and CO overpasses of New York City on November 6th, 2021, and Delhi, India on December 22nd, 2021, respectively. The urban domain used to analyze observations for each city is shown by the black square and world borders are shown. Pixels with warmer colors represent measurement of higher dry column mixing ratios and pixels are gray where there is low data quality, notably over bodies of water, mountainous regions, or are cloudy. (*F* and *G*) show a time series of the enhancement ratio, CH_*4,enh*_:CO_*enh*_ [mol/mol], for New York City and Delhi, respectively, from 2019 through 2023. Seasonal patterns and seasonal gaps in measurements are observed (*SI Appendix*, section 6).

We use a space-based approach to quantify enhancement ratios and CH_4_ emissions for 92 cities around the world in 2023. The locations of cities studied are shown in Fig. 1*A*. Example TROPOMI CH_4_ and CO overpasses are shown for New York City, United States of America on November 6th, 2021 and for Delhi, India, on December 22nd, 2021 in Fig. 1*B*–*E*. From daily TROPOMI data, we calculate daily enhancement ratios if data quality filtering requirements are met (*Materials and Methods*). The daily enhancement ratios, CH_*4,enh*_:CO_*enh*_, are shown for New York City and Delhi in Fig. 1*F*–*G*. We calculate annual enhancement ratios and subsequently, annual CH_4_ emission estimates using the Emissions Database for Global Atmospheric Research (EDGAR) v8.1 CO emission inventory for 92 cities across 7 regions, including Central East Africa; Africa; East, Southeast Asia & Oceania; North America; Latin America; South & West Asia; and Europe. For 72 of the 92 cities examined in this study, there is sufficient data density to track how the enhancement ratios and CH_4_ emissions change in time from 2019 through 2023.

The observed changes in the urban CH_4_ emissions are driven primarily by changes in the observed CH_4_ enhancements. The enhancement ratio is strongly correlated with the CH_4_ enhancement (r = 0.84). Changes in the CO enhancement, an average increase of 5% from 2019 to 2023, are often smaller and less variable than changes in the CH_4_ enhancement, an average increase of 10% from 2019 to 2023. The changes in the CO enhancement over time show less correlation with changes in the enhancement ratio (r =−0.33). The CO enhancement reflects changes in transport that also impact CH_4_ in the urban domain as well as changes in urban CO emissions. If the observed changes in enhancements and enhancement ratio were due solely to a decline in urban CO emissions, an unrealistic, widespread reduction in urban ventilation, approximately 10% across 72 cities between 2019 and 2023, would have additionally occurred. Changing CO emissions, whether manifested in the inventory or not, are incorporated into our methodology and uncertainty analysis as described in *Materials and Methods* and *SI Appendix*, section 4. Reported CO emissions do not drive the observed change in the CH_4_ emissions. Annual EDGAR v8.1 CO emissions in 2023 are, on average across cities, only 0.46% lower than in 2019, with a SD of 6.2%. Where the CO inventory does predict larger changes, the atmospheric CO changes commensurately leading to very small changes in inferred CH_4_ emissions. We highlight two examples, in Kyiv and Istanbul, in *SI Appendix*, section 5 where changes in the inferred CH_4_ emissions are not driven by the large changes (±20%) in the reported CO emissions. Additionally, our uncertainty analysis conservatively incorporates the impact of possible CO emissions changes not reflected in the inventory estimates. Thus, the method we use to infer annual CH_4_ emissions across urban areas and how they change over time accounts for, but is not governed by, changing CO emissions.

**Fig. 2.**
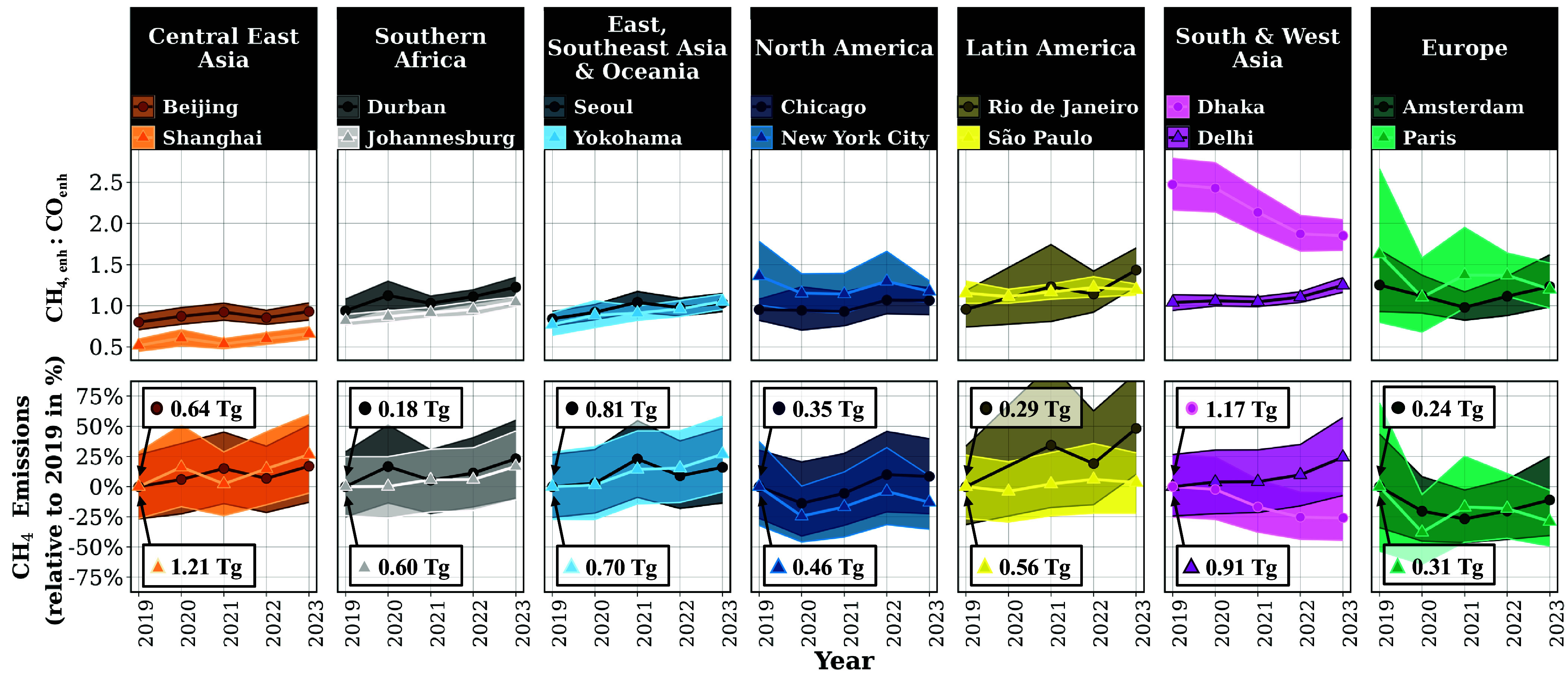
Annual enhancement ratios, CH_4,*enh*_:CO_*enh*_, and observed CH_4_ emissions for two cities in each C40 region. The cities selected are among the top contributors to the region’s urban CH_4_ emissions. The annual enhancement ratios and annual CH_4_ emissions show variability in time from 2019 to 2023 and are different across cities. The observed CH_4_ emissions are shown as percentage relative to 2019 values. The 2019 CH_4_ emissions are written to anchor the relative percent values. The shaded region of the annual enhancement ratios shows the 95%CI derived from instrumental, retrieval, and methodological uncertainty. The shaded region of the annual CH_4_ emissions show the 95%CI derived from the propagation of the uncertainty on the annual enhancement ratio with the uncertainty of how CO emissions change over time.

There are regional differences in observed annual enhancement ratios and CH_4_ emissions. We highlight the annual enhancement ratios and CH_4_ emissions for a subset of cities from 2019 to 2023 in Fig. 2. The cities shown are among the top contributors to their region’s total CH_4_ emissions. Average annual enhancement ratios are often near 1.0, but tend to be lower across Central East Asia due to strong CO enhancements. The observed CH_4_ emissions for each city are shown in the *Bottom* row of Fig. 2 as a percentage relative to 2019 emissions for that city. Although a bottom–up CO estimate is used to translate the enhancement ratio to a CH_4_ emission rate, the observed emissions trajectory from 2019 to 2023 is driven by the enhancement ratios. Many cities, including Durban, Johannesburg, Beijing, Shanghai, Yokohama, Seoul, Rio de Janeiro, São Paulo, and Delhi, show increasing enhancement ratios and CH_4_ emissions. Some cities have wider CIs driven by data availability and variability, making changing annual CH_4_ emissions insignificant. In such cases, enhancement ratios and emissions remain statistically unchanging, as seen in the European and North American cities in Fig. 2. Dhaka in South & West Asia exhibits a statistically decreasing enhancement ratio with an apparent but insignificant decrease in CH_4_ emissions. Most regions include a majority of cities with stagnant or increasing emissions, with the exception of Europe, with many cities showing stagnant to slowly decreasing emissions. A comprehensive table of the observed annual enhancement ratios, CH_4_ emissions, and respective CIs for all cities studied is shown in *SI Appendix*, Table S1.

**Fig. 3.**
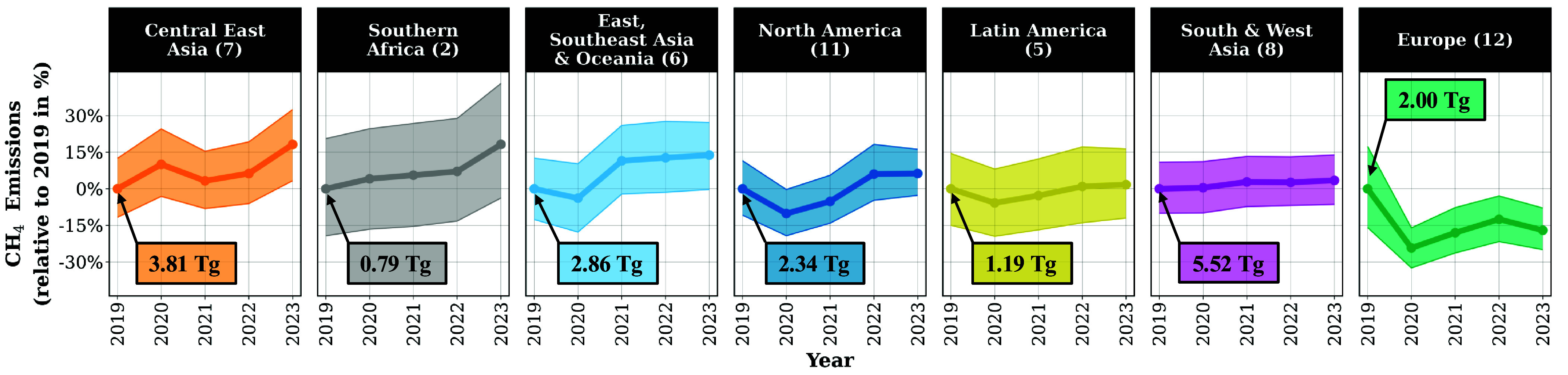
Regional sums of observed CH_4_ emissions [Tg/y] for C40 cities. The sums are shown as percent relative to their 2019 levels to highlight change over time. Only Europe shows decreasing emissions. The shaded region shows the 95%CI in detection of change over time. Note that annual observations across 2019 to 2023 are available for only 2 C40 cities in Africa, which are both located in Southern Africa.

We see broadly stagnant or increasing regionally summed annual CH_4_ emissions across 6 of the 7 C40 regions from 2019 through 2023. Aggregating into regional sums yields clearer detection of changing emissions over the study period, demonstrated by the regional urban CH_4_ emissions in Fig. 3. Distinct variations across years in the regionally summed CH_4_ emissions are evident even though all cities may not show statistically significant changes when examined individually. Additionally, there are regional differences in how annual CH_4_ emissions change. We assess the confidence in the detection of emission changes at two levels, 95% and 90%, (95%CI) and (90%CI) respectively, (*Materials and Methods* and *SI Appendix*, section S4). If the CI describing the difference in emissions between two years of interest excludes 0, there is a statistically significant difference between emissions at that confidence level. We observe an increase in CH_4_ emissions in Central East Asia, significant to the 90%CI, of 0.69 Tg (95%CI: −0.02, 1.40 Tg; 90%CI: 0.10, 1.28 Tg) from 2023 to 2019. The annual CH_4_ emissions from both East, Southeast Asia & Oceania and North America show an insignificant decrease from 2019 to 2020 followed by significant increases of 0.50 Tg (95%CI: −0.07, 1.07 Tg; 90%CI: 0.03, 0.99 Tg) and 0.38 Tg (95%CI: 0.07, 0.71 Tg; 90%CI: 0.12, 0.65 Tg) from 2020 to 2023. Southern Africa and Latin America exhibit gradually, insignificantly increasing emissions, while the CH_4_ emissions of South & West Asia appear stagnant. Europe shows an overall decrease in CH_4_ emissions almost entirely due to a significant decline in emissions from 2019 to 2020 of −0.48 Tg (95%CI: −0.89, −0.11 Tg; 90%CI: −0.82, −0.17 Tg). More than half of the European cities show gradually decreasing emissions. For example, Amsterdam’s annual emissions in Fig. 2 drop in 2020, although remaining within the CI. Aggregation of European city emissions show decreasing emissions from 2019 to 2023 that are robustly detectable at the regional level to the 90%CI (−0.34 Tg; 95%CI: −0.74, 0.03 Tg; 90%CI: −0.67, −0.03 Tg). Nevertheless, there are cities with stagnant and even increasing emissions in Europe. The converse scenario is true as well; regions that have increasing emission include cities with stagnant or decreasing emissions. Declining emissions in C40 cities are overshadowed because most cities, especially those with the largest contribution to regional aggregation of emissions, have stagnant or increasing emissions.

**Fig. 4.**
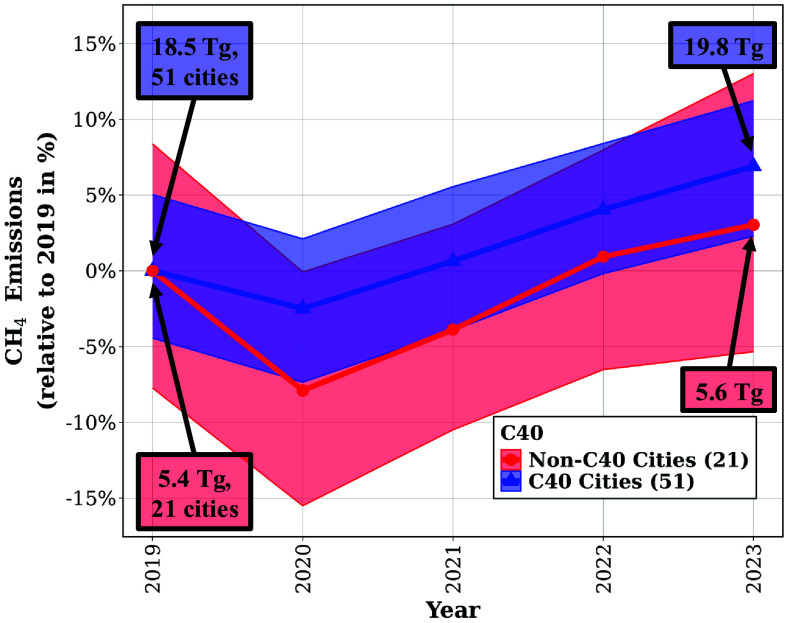
Observed summed CH_4_ emissions [Tg/y] for 51 C40 and 21 non-C40 cities. The sums are shown as percent relative to their 2019 levels. The shaded region shows the 95%CI in detection of change over time.

C40 cities in aggregate show a statistically significant increase in CH_4_ emissions from 2020 to 2023 at both the 95%CI and 90%CI. This corresponds to an emission increase of 1.72 Tg (95%CI: 0.38, 3.10 Tg; 90%CI: 0.58, 2.85 Tg), or 10% higher than in 2020 (95%CI: 2%, 17%; 90%CI: 3%, 16%). We also observe an increase, significant to the 90%CI, in CH_4_ emissions of non-C40 cities between 2020 and 2023, corresponding to an increase of 0.59 Tg (95%CI: −0.07, 1.25 Tg; 90%CI: 0.03, 1.15 Tg), or 12% higher (95%CI: −1.5%, 25%; 90%CI: 0.6%, 23%). Both groups of cities experience a statistically insignificant decline in CH_4_ emissions from 2019 to 2020. Fig. 4 compares total CH_4_ emissions of 51 C40 cities with 21 non-C40 cities. C40 cities emitted 18.5 Tg CH_4_ in 2019, with a decrease to 18.0 Tg in 2020, then rising to 19.8 Tg CH_4_ in 2023. The 21 non-C40 cities emitted 5.4 Tg CH_4_ in 2019, decreasing to 5.0 Tg CH_4_ in 2020 and increasing to 5.6 Tg in 2023. The 2020 emissions decrease was more pronounced in non-C40 cities, potentially due to having fewer cities in this aggregation. We theorize on the change in 2020 further in *SI Appendix*, section 8.

The aggregate CH_4_ emissions of all 72 cities (51 C40, 21 non-C40) exhibit a significant increase at the 90%CI of 1.40 Tg (95%CI: −0.08, 2.94 Tg; 90%CI: 0.17, 2.70 Tg) or a 6% change from 2019 to 2023. Direct comparison of emissions in 2020 and 2023 indicate a significant, larger increase at both 95%CI and 90%CI of 2.32 Tg (95%CI: 0.82, 3.85 Tg; 90%CI: 1.04, 3.61 Tg) or a 10% change, following an insignificant decrease in total emissions from 2019 to 2020. In total across the 72 continuously monitored cities, we observe the annual CH_4_ emissions drop from 23.9 Tg in 2019 to 23.0 Tg in 2020, followed by increases to 23.8 Tg in 2021, 24.7 Tg in 2022, and 25.3 Tg in 2023. The total annual CH_4_ emissions for all aggregate groupings are listed in *SI Appendix*, Table S2.

Our observations demonstrate increasing urban CH_4_ emissions, disagreeing with the stagnant bottom–up urban CH_4_ emissions shown across versions of EDGAR. We compare our observations to CH_4_ over the same domains in EDGAR v7 (released in 2021), v8 (released in 2022), and v2024 (released in 2024). In 2021, we observe total emissions among the 83 cities for which we have data to be 25.6 Tg CH_4_ (95%CI: 18.1, 33.2), where cumulative emissions of these cities is reported to be 21.3 Tg in EDGAR v2024, 26.0 Tg in EDGAR v8, and 23.4 Tg in EDGAR v7. Urban methane emission sources vary by city and region, with EDGAR v8.1 reporting the largest emissions from waste, agriculture, and energy sources as shown in *SI Appendix*, Fig. S3. The accuracy of this sectoral distribution is not established (*SI Appendix*, section 2). The changes across versions of EDGAR are within the error range suggested by Mastrogiacomo et al. (34) for EDGAR v8 CH_4_ over urban areas (34%), the fluctuation in CH_4_ estimates across EDGAR versions for the same domains suggests a lack of certainty in bottom–up characterization of urban emissions. We find that EDGAR v8 is in better agreement with our 2021 aggregated global emission estimate compared to other versions of the EDGAR CH_4_ inventory. However, while the EDGAR v8 magnitude is comparable with the total urban emissions across cities for a single year of observations, EDGAR v8 and other versions report near-constant emissions trajectories in most cities in contrast with our observations. Thus, our observations do not agree with EDGAR for the entire period of study. We see better agreement in the emissions growth in the most recent version, v2024. We observe CH_4_ emissions in 2023 over the 72 cities to be 6% larger than in 2019 and 10% larger than in 2020. EDGAR v8 CH_4_, which includes emissions through 2022, reports the total change from 2019 to 2022 as 3% and the 2020 to 2022 change as 1.7%, while EDGAR v2024 CH_4_ reports 5% for the same cities for the change between 2019 and 2023 and 3.7% for the change between 2020 and 2023. If EDGAR v8 continued at the same rate reported in previous years past 2022, the total change would still be lower than what is seen in both EDGAR v2024 and observations. There are differences between behavior of observed emissions from 2019 to 2020 compared the inventories and disagreement in the magnitude of change over the time period that both the observations and inventory exhibit increase from 2020 to 2023. Inconsistencies across inventory versions in urban CH_4_ emissions magnitude and change over time points to the critical role of observations to evaluate bottom–up inventories, especially as they are used in mitigation planning.

## Discussion

Urban environments’ role in the global CH_4_ budget was previously unquantified. In 2023, we find the urban contribution from 92 cities, some of the largest or most densely populated in the world and representing 1.18 billion people, to be 31.2 Tg CH_4_/y (95%CI: 22.3, 40.4 Tg CH_4_/y). This is equivalent to ∼10% of the total global anthropogenic methane budget (5). Urban areas present significant opportunities for emission reductions with this total contribution to the global budget, yet are studied less often compared to oil and gas basins. The 31.2 Tg in urban CH_4_ emissions is 3.75 times greater than the total contribution of oil and gas “Ultra-Emitters” reported in Lauvaux et al. (48). Further, urban areas represent unique combinations of various sectors and sources. Quantifying total city emissions provides an important backdrop for sector- and facility-specific work to quantify, monitor, and reduce CH_4_ emissions. Additionally, quantifying total city emissions aligns with emissions reduction planning and commitments like C40, which are also done at the city-scale.

Although the magnitude of urban emissions is substantial compared to the global budget, the total increase of 2.3 Tg CH_4_ since 2020 would explain only a fraction of the recent atmospheric methane surge (49). Urban areas are complex combinations of CH_4_ emission sources with thermogenic and biogenic signatures and quantifying both the total contribution of these areas and how they change over time adds additional constraint to our understanding of atmospheric CH_4_. Further, urbanization in the tropics may lead to increasing urban emissions which will need to be considered when assessing possible growth in tropical CH_4_ emissions. The observed increase across cities underscores the importance of monitoring urban emissions for effective planning and implementation of reduction strategies and for constraining understanding of atmospheric CH_4_ changes.

Considering the C40 city network’s commitments to reduce emissions, we expect to see differences in the trajectory of CH_4_ emissions. Interestingly, CH_4_ emissions increased regardless of C40 participation. While some cities and regions exhibit decreases, similar patterns are seen in non-C40 cities, indicating a broad rise in total emissions across both groups. Despite setting clear standards for reporting, C40 leadership does not verify reported city emissions. There is overlap of city participation in initiatives such as Local Governments for Sustainability (ICLEI) (50), the Global Covenant of Mayors for Climate and Energy (51), and the Race to Zero (7). Categorizing cities by these pledges does not provide a stronger correlation to explain the increase in emissions. Additionally, C40 cities are up to 3 times more likely to be involved in additional climate-related commitments compared to the non-C40 cities studied here. Thirty-three C40 cities studied additionally participate in all three of these other commitments but we have yet to observe a discernible impact of these commitments.

The C40 city network now faces the challenge of accounting for nearly 2 additional Tg of CH_4_ per year in addition to reducing emissions by 6 Tg/y as pledged by 2030. This 6 Tg/y reduction pledge emerges from the C40 network requirement to join the Cities Race to Zero. The Cities Race to Zero is a city-specific pathway in the Race to Zero, which assembles the largest ever alliance of actors committed to achieving net zero carbon emissions by 2050, at the latest. Race to Zero calls for a methane-specific emissions reduction of 34% by 2030, presumably from a 2020 baseline when the commitment was founded (7), which we calculate to be reducing annual emissions by 6.1 Tg/y. Urban methane emissions of C40 cities have increased by 1.7 Tg since the commitment was initiated in 2020. More ambitiously, C40 encourages cities to reduce methane emissions by 50% by 2030 (8), necessitating more substantial action and even faster reduction rates. The CH_4_ emissions reduction plans for C40 cities focus on moving away from greenhouse gas-powered electricity sources; optimizing energy use, as many have found leaks of natural gas may be correlated with consumption; increasing efficiency of landfill gas collection systems; decreasing the amount of waste sent to landfill; and improving landfill cover management (52, 53). On-going space-based monitoring of urban emissions would be able to detect a 34% to 50% reduction in urban CH_4_ emissions from the entire C40 network by 2030. If measures have already been taken by cities, their efficacy must be critically evaluated. However, it is possible that many mitigation measures have yet to be implemented as C40’s more specific initiatives for reducing methane emissions of the waste sector were founded in 2022 (9), leaving little time to observe the full impact. Across the coming decades, integration of satellites including target and area mappers will be vital in the multilevel monitoring efforts needed to quantify emissions, evaluate bottom–up emissions reports from the city level, and monitor the trajectory of emissions over time.

## Materials and Methods

### Defining an Urban Domain.

In this study, we isolate 92 cities across the world to quantify CH_4_ emissions. These cities represent many of the largest or most densely populated cities across the world. We compare the CH_4_ emissions trajectories for cities in the C40 network to a set of non-C40 cities to evaluate the connection between an emissions reduction pledge and changing fluxes. The locations of cities studied are shown in Fig. 1*A*.

For consistency across regions, urban domains in this work are defined by a box centered on the peak in the city population density from the NASA Gridded Population of the World (version 4) (54). An algorithm explained in *SI Appendix*, section 2 is used to isolate where the box has captured the city’s population by mapping the average population density of the domain decreasing as the area is expanded. The domain is always square, yet the size is specific to each city. Plant et al. (25) showed that the enhancement ratio is fairly insensitive to domain definition, as long as the dense core is incorporated. The domain selection used here ensures capture of this core region and establishes a uniform methodology across regions to utilize the characteristic relationship between the two trace gases at the urban level. Subsequently, the urban domain is always larger than the city’s borders, encapsulating the suburban and larger metropolitan regions, however, the municipalities used to name each region are responsible for a large portion of the population and inventoried emissions for each urban domain. For clarity in reference to the C40 network, we will refer to the areas described as cities, though surrounding urban areas will be included. Additionally we show that the annual enhancement ratios are not highly sensitive to changing the domain size. Closely neighboring cities are combined into one urban domain, resulting in 91 independent C40 cities to be evaluated. We do not have observations across all of these cities, however, due to data quality or filtering requirements discussed below. The urban domain of cities located along a coastal boundary are expanded 25% to account for the fraction of the domain taken up by water. *SI Appendix*, Table S1 lists the city names, locations including C40-designated region, and urban domain size. Further explanation of the urban domain including a sectoral breakdown of reported CH_4_ emissions, a list of combined neighboring cities, and sensitivity study of selection and adjustment of box size is located in *SI Appendix*, section 2.

### Satellite Observations.

This work utilizes Level 2 observations of CH_4_ and CO from January 2019 through December 2023 from the TROPOspheric Monitoring Instrument (TROPOMI) on board the Sentinel-5 Precursor satellite (46, 47, 55, 56). TROPOMI, launched in October 2017, has daily global coverage at 13:30 local solar time with a resolution of 5.5 km × 7 km since August 2019, previously 7 km × 7 km (57). The TROPOMI CH_4_ and CO retrievals are based on the observed reflected solar radiation in the shortwave infrared spectrum. An example of a TROPOMI CH_4_ and CO overpass for New York City, United States of America and for Delhi, India is shown in Fig. 1*B*–*E*. The CH_4_ product is the bias-corrected dry column mixing ratio while the CO product is the column density, which we convert to a dry column mixing ratio using provided surface pressure. *SI Appendix*, section 3 shares additional details regarding the TROPOMI data products used.

We filter the TROPOMI overpasses according to recommendations for data usage provided with the TROPOMI operational data (58, 59) as well as Plant et al. (25), removing overpasses with high aerosol loads or clouds, removing pixels over water, and disregarding overpasses when there is no Visible Infrared Imaging Radiometer Suite (VIIRS) based cloud cover data. We select the TROPOMI CH_4_ and CO pixels found within the city’s urban domain In addition to filtering for observation quality, we consider only CH_4_ and CO pixels that are colocated, or spanning the same grid space. We require these colocated pixels to cover at least 20% of the urban domain by area. After this colocation check, we filter out days impacted by nearby wildfire CO emissions following Leguijt et al. (60, 61).

### Quantification Methods.

We use a tracer–tracer approach where CH_4_ and CO are emitted from the urban area, leading to elevated observed values of both gases above and downwind from the urban center. We do not consider chemical loss of either species as they are both long-lived relative to the urban domain scale. We use the distribution of column mixing ratios within the prescribed urban domain to derive a background value for each overpass as a low percentile (15th) of the daily values. This 15th percentile reduces the influence of spuriously low retrievals and prevents further data loss that would occur if a different background method was utilized. Further details are provided in Plant et al. (25). Subtracting the background yields the daily CH_4_ and CO enhancement values ($\Delta CH_{4},\Delta \mathrm{CO}$). We scale the enhancements with the respective averaging kernel closest to the surface ($A_{0}$) and account for bias introduced by enhancements in the a priori column ($prior_{\mathrm{enh}}$) shown in Eqs. **1** and **2** (45):

[1] $$\begin{array}{c} {\left(CH_{4}\right)}_{\mathrm{enh}}=\frac{\Delta \;CH_{4}}{A_{0,\;CH_{4}}}\;-\;\frac{\left(1\;-\;A_{0,\;CH_{4}}\right)\left(prior_{enh,\;CH_{4}}\right)}{A_{0,\;CH_{4}}} \end{array}$$

[2] $$\begin{array}{c} {\left(CO\right)}_{\mathrm{enh}}=\frac{\Delta \;CO}{A_{0,\;CO}}\;-\;\frac{\left(1\;-\;A_{0,\;CO}\right)\left(prior_{enh,\;CO}\right)}{A_{0,\;CO}} \end{array}$$

We then sum the enhancements from each pixel across the selected domain resulting in the daily ratio of summed enhancements CH_*4,enh*_:CO_*enh*_, or enhancement ratio:

[3] $$\begin{array}{c} \frac{\Sigma {\left(CH_{4}\right)}_{\mathrm{enh}}}{\Sigma {\left(CO\right)}_{\mathrm{enh}}} \end{array}$$

This enhancement ratio reflects an integration across the domain as a whole. This approach is distinct from other tracer–tracer applications and does not require the tracers to be coemitted or for enhancements to be spatially correlated (25). The daily ratio is further filtered to reduce bias when the enhancement-to-noise ratio in the denominator of [**3**] is close to unity, therefore the CO enhancement filtering threshold is 7 ppb/pixel following Plant et al. (25). The enhancement ratios are entirely data-driven–they do not depend on modeling or reported emissions inventories. This means the ratios are robust, independent, daily observables that can be tracked, providing insight into changes to each city’s CH_4_:CO relationship. We compute the annual average from the daily enhancement ratios, requiring each city to have at least 5 overpasses in a year. To characterize the uncertainty on the annual enhancement ratio, we employ a Monte-Carlo approach by varying the background percentile and specific retrieval parameters to add noise to the retrieved TROPOMI CH_4_ and CO column mixing ratios. This process produces a distribution of possible annual enhancement ratio realizations, reflecting data variability, impact of background percentile choice, and uncertainty in the measurement and retrieval. More details on the uncertainty methodology are provided in *SI Appendix*, section 4.

Urban sources of CO are tethered to fossil fuel combustion and are generally better captured by inventories compared to CH_4_. The sources of urban CH_4_ are more varied and large discrepancies have been shown in observed urban emissions compared to inventory estimates (e.g. refs. 16, 19, 22, 23, 27, and 28). We therefore use the CH_*4,enh*_:CO_*enh*_ ratio to relate the known CO emissions to the city’s CH_4_ emissions without transport modeling:

[4] $$\begin{array}{c} E_{CH_{4}}\;=\;\frac{\Sigma {\left(CH_{4}\right)}_{\mathrm{enh}}}{\Sigma {\left(CO\right)}_{\mathrm{enh}}}\;\times \;\frac{M_{CH_{4}}}{M_{\mathrm{CO}}}\;\times \;E_{\mathrm{CO}} \end{array}$$

To calculate the CH_4_ emissions (E*_CH_4__*), we multiply the enhancement ratio by the emissions of CO reported in an inventory (E_*CO*_) and by the ratio of the molecular masses of CH_4_ and CO (M_*CH_4_*_/M_*CO*_) to account for the mass difference. We then can interrogate patterns and changes in both the enhancement ratios and the emissions. When calculating a regional or total sum of emissions across cities, only cities with data for every year 2019 to 2023 are used. We use the Emissions Database for Global Atmospheric Research Version 8.1 annual CO gridded inventory (EDGAR v8.1 CO), released in 2024, for the conversion of the ratio between the two gases to emissions of CH_4_ (62–64). Additionally, we compare the quantification of CH_4_ emissions to the CH_4_ emissions reported in the EDGAR Version 8.0 annual CH_4_ gridded inventory (EDGAR v8 CH_4_) (63, 65, 66), released in 2023 as well as in the EDGAR Version 2024 annual CH_4_ gridded inventory (EDGAR 2024 CH_4_) (67, 68) to examine how well the global inventory represents CH_4_ emissions from urban areas. The native EDGAR grid is 0.1° × 0.1° and the inventory emissions are calculated for the same spatial domain as the TROPOMI observations. The TROPOMI dataset used here includes observations in 2023, so we use the 2022 value of the EDGAR CO and CH_4_ inventories in place of 2023 if the dataset ends earlier. Further explanation of CO inventory use is located in *SI Appendix*, section 5.

We implement two different methods for calculating uncertainty: one to represent the absolute uncertainty on the total CH_4_ emissions and one to represent uncertainty on the detection of change in inferred annual CH_4_ emissions over time. Both uncertainty methodologies are outlined here and further discussed in *SI Appendix*, section 4. Determining the absolute uncertainty on the total CH_4_ emissions relies on the knowledge of the absolute emissions of CO. While the bottom–up inventories for CO emissions are often more accurate than the bottom–up inventories of CH_4_ emissions, there are discrepancies with observations as shown in Leguijt et al. (60). Accordingly, uncertainty of inventory values, if provided, are often very high. Wide CIs on absolute emissions reflect confidence in the overall magnitude rather than the change over time. The absolute EDGAR CO uncertainty is derived from regional uncertainties listed in *SI Appendix* of Crippa et al. (69). The dependence on the CO inventory in this method is most impactful in the calculation of the total magnitude of CH_4_ emissions, rather than their relative change over time as observations suggest urban CO emissions have insignificant change over years (60).

The uncertainty reflecting confidence in the detection of change in CH_4_ emissions is distinct from the absolute uncertainty on the CH_4_ emissions and must account for changes in CO emissions over time. We explicitly include any annual changes in CO emissions captured by the CO inventory in our analysis through direct multiplication with the observed enhancement ratios. We additionally incorporate possible changes in CO emissions not accounted for in the EDGAR inventory through our calculation of CIs of the annual CH_4_ emissions. We derive uncertainty from an independent, observational study of urban CO emissions from 2019 to 2021 that used a cross-sectional flux (CSF) approach following Leguijt et al. (60). This CSF study found declining urban CO emissions, 12% over two years, on average across the dense urban cores over 67 cities worldwide, 43 of which are also observed in this work. The urban cores described by the CSF study estimates are much smaller than the total urban area domains used in this work. A global analysis suggests total anthropogenic CO emissions decline 0.69(±0.27) to 0.7(±0.26) %/y from 2000 to 2017 (70). The change of −12% over 2 y within the urban cores is unlikely to occur across the entire spatial domain of each city and also in each city across the world, universally. We conservatively incorporate uncertainty of unknown CO emissions changes by imposing Gaussian noise (σ=12%/y) on the EDGAR v8.1 CO emissions for each city, without assuming direction. When translating the annual enhancement ratios to annual CH_4_ emissions, we randomly sample from this annual CO emissions distribution so that both known and potential unknown changes in annual urban CO emissions are accounted for within the resulting means, CIs, and detection of changing CH_4_ emissions over time. In *Results*, we demonstrate how the observed changes in annual CH_4_ emissions are robust to possible changes in annual CO emissions and further discuss the CSF study and the incorporated possibly changing CO emissions in *SI Appendix*, section 4.

By incorporating a distribution of annual enhancement ratios and a distribution of annual CO emissions in Eq. **4**, we obtain a distribution of annual CH_4_ emissions for each city and year that includes uncertainty from both the observed enhancements and the CO emissions. We find regional and total emissions in aggregate by summing the randomized annual distributions, yielding an emission distribution for the specified level of aggregation, from which a respective mean and CI can be computed. To meaningfully speak to the detection of difference between years, we use a Monte-Carlo approach to formulate a distribution representing the difference between annual CH_4_ emissions of different years at the regional and total scales. From the resulting CH_4_ emission distributions, we assess the mean change in CH_4_ emissions and the statistical significance of observed changes between years at both the 95% and 90% confidence levels. This method is described in more detail in *SI Appendix*, section 4, and allows us to assess if observed emission changes are statistically robust.

## Supplementary Material

Appendix 01 (PDF)

## Data Availability

All code used in data processing and analysis for our study is available in the Zenodo repository at https://doi.org/10.5281/zenodo.17582608 (71). The TROPOMI Level 2 CO and CH_4_ data at 5.5 km × 7 km resolution used in this study are openly available from NASA Earth Data at https://search.earthdata.nasa.gov/ (55, 56). Alternatively, both datasets are openly accessible at https://sentiwiki.copernicus.eu/web/s5p-products (46). This study was generated using Copernicus Atmosphere Monitoring Service Information (2022) to filter out overpasses impacted by wildfire CO. The variable “Wildfire flux of carbon monoxide (CO)” was used from 2019-01-01 to 2023-12-31 and can be accessed at https://ads.atmosphere.copernicus.eu/datasets/cams-global-fire-emissions-gfas?tab=overview (72). The Gridded Population of the World, Version 4 (GPWv4) for year 2020, used to find the urban center of each city, is openly available at https://www.earthdata.nasa.gov/data/catalog/sedac-ciesin-sedac-gpwv4-popcount-r11-4.11 (54). Information about C40 cities and a complete list of current city membership is available at https://www.c40.org/cities/ (6). The C40 city population and area was powered by CDP Data (http://data.cdp.net), specifically in the Global Protocol for Community-scale inventory data for C40 cities. This reported dataset can be accessed on the C40 Cities Climate Leadership Group (2023) Greenhouse Gas Emissions Interactive Dashboard at https://www.c40knowledgehub.org/s/article/C40-cities-greenhouse-gas-emissions-interactive-dashboard?language=en_US or directly downloaded from CDP at https://drive.google.com/uc?id=1zAQy4GdVwssHFZRQs8pbPESEGGg_Aj3Q&export=download (73). The Global Human Settlement Layer Degree of Urbanization is available for 2020 at https://human-settlement.emergency.copernicus.eu/download.php?ds=smod (74). The EDGAR annual gridmaps from 2019 to the most recent year available are openly accessible for version 8.1 for CO: https://edgar.jrc.ec.europa.eu/index.php/dataset_ap81 (64); version 7 for CH_4_: https://edgar.jrc.ec.europa.eu/dataset_ghg70 (75); version 8 for CH_4_: http://data.europa.eu/89h/b54d8149-2864-4fb9-96b9-5fd3a020c224 (66); and version 2024 for CH_4_: https://edgar.jrc.ec.europa.eu/dataset_ghg2024 (68). Note that the EDGAR v8.1 CO and EDGAR v8 CH_4_ files used in this study were redownloaded to encompass updates to emissions in September 2024. We have noticed announced and unannounced changes to the emissions in the files, see *SI Appendix*, section 5 for more on this. The HTAPv3 annual grid maps for CO in 2018 are openly available at https://edgar.jrc.ec.europa.eu/dataset_htap_v3 (76).
