# Supplementary material for: Space-based observation of global increase in urban methane emissions from 2019–2023
Source: Proc Natl Acad Sci U S A. 2026 Apr 13;123(16):e2504211123. doi: 10.1073/pnas.2504211123 (PMC13099624; doi:10.1073/pnas.2504211123)
Supplement: Supplementary file 1 — Appendix 01 (PDF) [file pnas.2504211123.sapp.pdf]

PNAS

Supporting Information for

Space-based observation of global increase in urban methane emissions from 2019-2023

Erica Whiting, Genevieve Plant, Eric A. Kort, Ilse Aben, Kira J. Biener, Gijs Leguijt, Joannes D. Maasakkers

Corresponding Author
E-mail: erwh@umich.edu or eric.kort@mpic.de

This PDF file includes:

Figs. S1 to S13
Tables S1 to S5
SI References

Contents

1 Emissions Tables 3

2 Urban Domain Selection 12

3 TROPOMI Data 14

4 Uncertainty on the Annual Enhancement Ratio and Annual Emissions 15
A Uncertainty Methodology . . . 15
B Interpretation of Change . . . 19

5 CO Inventory 20
A EDGAR CO v8.1 . . . 20
B Case Studies of Large EDGAR CO v8.1 Change: Kyiv and Istanbul . . . 20
C Shanghai, China . . . 20
D Other CO Inventories . . . 21

6 Seasonal Emissions 21
A Seasonal CH_4 Emissions . . . 21
B Seasonal CO Emissions . . . 22

7 Comparing City Emissions Across Methods 22

8 Investigating Change in Emissions in 2020 23

1. Emissions Tables

Table S1. CH_4:CO and CH_4 Emissions Data. The city name, country, C40-designated region, longitude, latitude, and urban domain size are listed. Non-C40 cities are assigned to a C40 region. The urban domain sizing is the distance in degrees from the city center to the box-edge latitude-wise and is further explained in SI S2. The number of observations that meet filtering requirements is noted, along with annual average summed enhancement ratio (CH_4:CO). The observed CH_4 emissions and reported CH_4 emissions in EDGAR v8 and v2024 are listed in Tg for each year available. The values in parenthesis show the 95% confidence intervals, rounded to two significant figures, that incorporate the uncertainty on the detection of change (see SI S4). EDGAR v8 CH_4 emissions data for 2023 is marked by NA because the data set contains emissions through 2022. All other NAs represent a year with no observations that meet filtering requirements.

Name, Region, Location	C40	Box	Type	2019	2020	2021	2022	2023
Addis Ababa, Ethiopia	Yes	0.64°	# Obs	0	0	0	0	7
Africa			CH4:CO	NA	NA	NA	NA	1.21 (0.84, 1.58)
(9.03°, 38.72°)			CH4 [Tg]	NA	NA	NA	NA	0.92 (0.58, 1.33)
			EDGARv8 CH4 [Tg]	0.65	0.67	0.69	0.71	NA
			EDGARv2024 CH4 [Tg]	0.52	0.54	0.55	0.56	0.56
Ahmedabad, India	Yes	0.65°	# Obs	23	44	56	62	69
South & West Asia			CH4:CO	0.96 (0.83, 1.1)	0.89 (0.8, 1)	0.96 (0.88, 1.05)	0.97 (0.9, 1.04)	1.05 (0.98, 1.13)
(23.01°, 72.55°)			CH4 [Tg]	0.4 (0.3, 0.53)	0.39 (0.29, 0.49)	0.42 (0.31, 0.52)	0.43 (0.32, 0.54)	0.47 (0.35, 0.59)
			EDGARv8 CH4 [Tg]	0.47	0.48	0.48	0.49	NA
			EDGARv2024 CH4 [Tg]	0.33	0.33	0.33	0.33	0.34
Amman, Jordan	Yes	0.51°	# Obs	0	6	10	17	23
South & West Asia			CH4:CO	NA	0.9 (0.7, 1.12)	1.03 (0.86, 1.2)	1.3 (1.14, 1.5)	1.2 (1.06, 1.35)
(31.99°, 35.99°)			CH4 [Tg]	NA	0.03 (0.02, 0.05)	0.04 (0.03, 0.06)	0.06 (0.04, 0.07)	0.05 (0.04, 0.06)
			EDGARv8 CH4 [Tg]	0.17	0.17	0.17	0.18	NA
			EDGARv2024 CH4 [Tg]	0.14	0.14	0.15	0.15	0.15
Amsterdam, The Netherlands	Yes	0.6°	# Obs	8	11	12	13	24
Europe			CH4:CO	1.25 (0.93, 1.68)	1.12 (0.91, 1.37)	0.98 (0.82, 1.17)	1.11 (0.88, 1.36)	1.23 (0.99, 1.62)
(52.37°, 4.87°)			CH4 [Tg]	0.24 (0.16, 0.35)	0.19 (0.13, 0.26)	0.18 (0.13, 0.24)	0.19 (0.14, 0.26)	0.22 (0.14, 0.3)
			EDGARv8 CH4 [Tg]	0.2	0.19	0.19	0.19	NA
			EDGARv2024 CH4 [Tg]	0.18	0.17	0.17	0.17	0.17
Asunción, Paraguay	No	0.61°	# Obs	46	32	70	63	68
Latin America			CH4:CO	1.04 (0.92, 1.19)	0.94 (0.85, 1.03)	1.03 (0.97, 1.09)	0.93 (0.87, 0.99)	1.04 (0.97, 1.13)
(-25.27°, -57.5°)			CH4 [Tg]	0.15 (0.11, 0.19)	0.13 (0.1, 0.17)	0.15 (0.11, 0.19)	0.14 (0.1, 0.17)	0.15 (0.11, 0.19)
			EDGARv8 CH4 [Tg]	0.09	0.1	0.1	0.1	NA
			EDGARv2024 CH4 [Tg]	0.08	0.08	0.08	0.08	0.08
Atlanta, USA	No	0.67°	# Obs	18	29	26	29	32
North America			CH4:CO	1.49 (1.04, 2.07)	0.93 (0.8, 1.07)	1.05 (0.9, 1.21)	0.97 (0.84, 1.12)	1 (0.88, 1.11)
(33.9°, -84.21°)			CH4 [Tg]	0.18 (0.11, 0.27)	0.1 (0.07, 0.13)	0.13 (0.09, 0.16)	0.12 (0.09, 0.15)	0.12 (0.09, 0.15)
			EDGARv8 CH4 [Tg]	0.12	0.12	0.12	0.12	NA
			EDGARv2024 CH4 [Tg]	0.11	0.11	0.11	0.11	0.11
Austin,TX, USA	Yes	0.55°	# Obs	0	9	8	20	14
North America			CH4:CO	NA	0.84 (0.59, 1.18)	0.99 (0.81, 1.18)	1.15 (1.01, 1.31)	1.23 (1.09, 1.35)
(30.29°, -97.75°)			CH4 [Tg]	NA	0.04 (0.03, 0.06)	0.05 (0.04, 0.07)	0.06 (0.05, 0.08)	0.07 (0.05, 0.08)
			EDGARv8 CH4 [Tg]	0.05	0.05	0.05	0.05	NA
			EDGARv2024 CH4 [Tg]	0.05	0.05	0.05	0.05	0.05
Baghdad, Iraq	No	0.64°	# Obs	17	27	23	27	49
South & West Asia			CH4:CO	0.85 (0.77, 0.95)	0.93 (0.84, 1.02)	1.06 (0.96, 1.17)	1.03 (0.91, 1.16)	1.08 (0.99, 1.18)
(33.44°, 44.53°)			CH4 [Tg]	0.03 (0.03, 0.04)	0.04 (0.03, 0.05)	0.04 (0.03, 0.06)	0.05 (0.03, 0.06)	0.05 (0.04, 0.06)
			EDGARv8 CH4 [Tg]	0.22	0.2	0.24	0.26	NA
			EDGARv2024 CH4 [Tg]	0.19	0.18	0.22	0.23	0.24
Bangkok, Thailand	Yes	0.7°	# Obs	31	23	35	20	29
East, Southeast Asia & Oceania			CH4:CO	1.08 (0.94, 1.24)	1.09 (0.82, 1.45)	1.13 (1.02, 1.26)	1.41 (1.22, 1.64)	1.25 (1.1, 1.4)
(13.72°, 100.54°)			CH4 [Tg]	0.73 (0.54, 0.94)	0.61 (0.42, 0.86)	0.63 (0.46, 0.8)	0.79 (0.58, 1.02)	0.7 (0.53, 0.9)
			EDGARv8 CH4 [Tg]	0.89	0.87	0.9	0.89	NA
			EDGARv2024 CH4 [Tg]	0.76	0.75	0.78	0.77	0.78

Table S1. CH_4:CO and CH_4 Emissions Data *(continued)*

Name, Region, Location	C40	Box	Type	2019	2020	2021	2022	2023
Barcelona, Spain	Yes	0.75°	# Obs	0	8	8	11	27
Europe			CH4:CO	NA	1.41 (1.16, 1.66)	1.9 (1.5, 2.42)	1.33 (1.16, 1.58)	1.62 (1.45, 1.8)
(41.38°, 2.15°)			CH4 [Tg]	NA	0.07 (0.05, 0.09)	0.1 (0.07, 0.13)	0.07 (0.05, 0.09)	0.08 (0.06, 0.11)
			EDGARv8 CH4 [Tg]	0.14	0.14	0.14	0.14	NA
			EDGARv2024 CH4 [Tg]	0.13	0.13	0.13	0.13	0.13
Beijing, China	Yes	0.59°	# Obs	62	57	41	48	48
Central East Asia			CH4:CO	0.8 (0.71, 0.9)	0.87 (0.78, 0.98)	0.92 (0.82, 1.03)	0.86 (0.77, 0.94)	0.93 (0.83, 1.03)
(39.86°, 116.24°)			CH4 [Tg]	0.64 (0.47, 0.81)	0.68 (0.5, 0.87)	0.74 (0.55, 0.94)	0.69 (0.51, 0.86)	0.75 (0.56, 0.97)
			EDGARv8 CH4 [Tg]	0.55	0.56	0.57	0.58	NA
			EDGARv2024 CH4 [Tg]	0.41	0.42	0.43	0.44	0.44
Bengaluru, India	Yes	0.55°	# Obs	10	12	24	24	36
South & West Asia			CH4:CO	0.82 (0.69, 1.05)	0.83 (0.67, 1.02)	0.86 (0.75, 0.97)	1 (0.9, 1.11)	0.97 (0.88, 1.07)
(12.96°, 77.55°)			CH4 [Tg]	0.34 (0.25, 0.48)	0.35 (0.25, 0.47)	0.37 (0.28, 0.48)	0.44 (0.33, 0.56)	0.42 (0.31, 0.54)
			EDGARv8 CH4 [Tg]	0.4	0.4	0.4	0.41	NA
			EDGARv2024 CH4 [Tg]	0.26	0.26	0.27	0.27	0.28
Berlin, Germany	Yes	0.57°	# Obs	6	20	16	14	31
Europe			CH4:CO	1.32 (0.82, 2.06)	1.13 (0.88, 1.51)	1.32 (1.08, 1.59)	1.04 (0.87, 1.22)	1.25 (1.03, 1.52)
(52.55°, 13.42°)			CH4 [Tg]	0.08 (0.04, 0.12)	0.06 (0.04, 0.09)	0.08 (0.05, 0.11)	0.06 (0.05, 0.08)	0.07 (0.05, 0.1)
			EDGARv8 CH4 [Tg]	0.09	0.09	0.09	0.09	NA
			EDGARv2024 CH4 [Tg]	0.09	0.08	0.08	0.08	0.08
Boston,MA, USA	Yes	0.56°	# Obs	20	18	22	23	24
North America			CH4:CO	1.07 (0.85, 1.29)	1 (0.77, 1.28)	0.99 (0.87, 1.11)	1.37 (1.05, 1.86)	1.13 (0.96, 1.28)
(42.35°, -71.09°)			CH4 [Tg]	0.16 (0.12, 0.22)	0.13 (0.09, 0.18)	0.15 (0.11, 0.19)	0.21 (0.14, 0.31)	0.17 (0.13, 0.22)
			EDGARv8 CH4 [Tg]	0.06	0.05	0.06	0.06	NA
			EDGARv2024 CH4 [Tg]	0.05	0.05	0.05	0.05	0.05
Brasilia,Brazil	No	0.72°	# Obs	11	24	22	27	23
Latin America			CH4:CO	1.23 (1.02, 1.49)	1.11 (0.96, 1.29)	1.16 (0.99, 1.36)	1.28 (1.17, 1.43)	1.28 (1.13, 1.43)
(-15.77°, -47.78°)			CH4 [Tg]	0.12 (0.09, 0.17)	0.11 (0.09, 0.15)	0.12 (0.09, 0.16)	0.14 (0.1, 0.17)	0.14 (0.1, 0.17)
			EDGARv8 CH4 [Tg]	0.13	0.13	0.13	0.13	NA
			EDGARv2024 CH4 [Tg]	0.11	0.11	0.11	0.11	0.11
Buenos Aires, Argentina	Yes	0.69°	# Obs	21	40	51	59	60
Latin America			CH4:CO	1.43 (1.17, 1.76)	1.37 (1.22, 1.54)	1.36 (1.25, 1.47)	1.31 (1.22, 1.4)	1.36 (1.25, 1.48)
(-34.6°, -58.4°)			CH4 [Tg]	0.16 (0.12, 0.22)	0.14 (0.1, 0.18)	0.15 (0.12, 0.19)	0.15 (0.11, 0.18)	0.15 (0.11, 0.19)
			EDGARv8 CH4 [Tg]	0.27	0.27	0.27	0.27	NA
			EDGARv2024 CH4 [Tg]	0.2	0.2	0.2	0.2	0.2
Cairo, Egypt	No	0.53°	# Obs	38	38	53	48	66
Africa			CH4:CO	1.37 (1.21, 1.57)	1.42 (1.24, 1.61)	1.35 (1.23, 1.47)	1.48 (1.33, 1.62)	1.49 (1.35, 1.63)
(30.08°, 31.2°)			CH4 [Tg]	0.65 (0.49, 0.81)	0.67 (0.5, 0.86)	0.67 (0.5, 0.84)	0.76 (0.55, 0.97)	0.77 (0.58, 0.96)
			EDGARv8 CH4 [Tg]	0.49	0.49	0.49	0.49	NA
			EDGARv2024 CH4 [Tg]	0.35	0.36	0.37	0.38	0.39
Calgary, Canada	No	0.58°	# Obs	17	19	26	22	36
North America			CH4:CO	1.03 (0.82, 1.26)	0.93 (0.78, 1.08)	0.9 (0.75, 1.07)	0.98 (0.88, 1.11)	1.26 (1.05, 1.48)
(51.05°, -114.09°)			CH4 [Tg]	0.04 (0.03, 0.05)	0.03 (0.02, 0.04)	0.03 (0.02, 0.04)	0.04 (0.03, 0.05)	0.05 (0.03, 0.06)
			EDGARv8 CH4 [Tg]	0.22	0.18	0.2	0.17	NA
			EDGARv2024 CH4 [Tg]	0.22	0.18	0.2	0.17	0.17
Cape Town, South Africa	Yes	0.78°	# Obs	0	0	0	0	10
Africa			CH4:CO	NA	NA	NA	NA	1.54 (1.27, 1.86)
(-34.02°, 18.66°)			CH4 [Tg]	NA	NA	NA	NA	0.29 (0.21, 0.39)
			EDGARv8 CH4 [Tg]	0.44	0.43	0.38	0.41	NA
			EDGARv2024 CH4 [Tg]	0.42	0.41	0.36	0.4	0.4
Chengdu, China	Yes	0.65°	# Obs	9	0	7	5	12
Central East Asia			CH4:CO	1.21 (1.03, 1.37)	NA	1.15 (0.89, 1.44)	1 (0.56, 1.38)	1.33 (1.07, 1.58)

Table S1. CH_4:CO and CH_4 Emissions Data *(continued)*

Name, Region, Location	C40	Box	Type	2019	2020	2021	2022	2023
(30.72°, 104.05°)			CH4 [Tg]	0.49 (0.36, 0.64)	NA	0.47 (0.33, 0.64)	0.41 (0.22, 0.6)	0.54 (0.38, 0.72)
			EDGARv8 CH4 [Tg]	0.55	0.55	0.56	0.56	NA
			EDGARv2024 CH4 [Tg]	0.46	0.47	0.47	0.47	0.48
Chennai, India	Yes	0.86°	# Obs	8	7	14	11	21
South & West Asia			CH4:CO	0.82 (0.64, 0.99)	1.03 (0.82, 1.26)	0.99 (0.9, 1.11)	1.13 (0.98, 1.28)	1.01 (0.9, 1.14)
(13.05°, 80.1°)			CH4 [Tg]	0.25 (0.17, 0.34)	0.32 (0.22, 0.43)	0.31 (0.23, 0.4)	0.36 (0.27, 0.46)	0.32 (0.25, 0.42)
			EDGARv8 CH4 [Tg]	0.22	0.22	0.23	0.22	NA
			EDGARv2024 CH4 [Tg]	0.17	0.17	0.18	0.17	0.18
Chicago,IL, USA	Yes	0.89°	# Obs	11	9	11	14	24
North America			CH4:CO	0.95 (0.82, 1.08)	0.94 (0.71, 1.23)	0.93 (0.76, 1.17)	1.07 (0.9, 1.28)	1.06 (0.89, 1.22)
(41.9°, -87.63°)			CH4 [Tg]	0.35 (0.26, 0.46)	0.3 (0.21, 0.42)	0.33 (0.24, 0.45)	0.39 (0.28, 0.51)	0.38 (0.27, 0.49)
			EDGARv8 CH4 [Tg]	0.27	0.27	0.27	0.27	NA
			EDGARv2024 CH4 [Tg]	0.26	0.26	0.26	0.26	0.26
Chongqing, China	No	0.6°	# Obs	7	6	8	7	12
Central East Asia			CH4:CO	1.33 (1.03, 1.76)	1.14 (0.88, 1.47)	1.14 (0.86, 1.43)	1.19 (0.95, 1.42)	0.91 (0.79, 1.04)
(29.55°, 106.55°)			CH4 [Tg]	0.57 (0.39, 0.81)	0.49 (0.34, 0.69)	0.51 (0.34, 0.7)	0.53 (0.37, 0.69)	0.4 (0.3, 0.52)
			EDGARv8 CH4 [Tg]	0.34	0.34	0.35	0.35	NA
			EDGARv2024 CH4 [Tg]	0.28	0.28	0.29	0.29	0.3
Copenhagen, Denmark	Yes	0.76°	# Obs	8	10	9	7	33
Europe			CH4:CO	1.7 (1.24, 2.2)	1.14 (0.79, 1.69)	1.24 (1.03, 1.56)	1.31 (0.95, 1.65)	1.2 (1.02, 1.43)
(55.67°, 12.55°)			CH4 [Tg]	0.07 (0.05, 0.1)	0.04 (0.03, 0.07)	0.05 (0.03, 0.06)	0.05 (0.03, 0.07)	0.05 (0.03, 0.06)
			EDGARv8 CH4 [Tg]	0.11	0.11	0.11	0.11	NA
			EDGARv2024 CH4 [Tg]	0.09	0.09	0.09	0.09	0.09
Curitiba, Brazil	Yes	0.53°	# Obs	26	42	34	20	43
Latin America			CH4:CO	1.12 (0.98, 1.28)	1.04 (0.93, 1.16)	1.02 (0.94, 1.11)	1.07 (0.94, 1.2)	1.08 (0.99, 1.18)
(-25.45°, -49.29°)			CH4 [Tg]	0.1 (0.08, 0.13)	0.1 (0.07, 0.12)	0.1 (0.08, 0.12)	0.1 (0.08, 0.13)	0.1 (0.08, 0.13)
			EDGARv8 CH4 [Tg]	0.16	0.16	0.16	0.16	NA
			EDGARv2024 CH4 [Tg]	0.12	0.13	0.13	0.13	0.13
Dalian, China	Yes	0.62°	# Obs	0	0	0	0	6
Central East Asia			CH4:CO	NA	NA	NA	NA	1.03 (0.77, 1.35)
(38.9°, 121.55°)			CH4 [Tg]	NA	NA	NA	NA	0.16 (0.11, 0.23)
			EDGARv8 CH4 [Tg]	0.12	0.12	0.13	0.13	NA
			EDGARv2024 CH4 [Tg]	0.09	0.09	0.1	0.1	0.1
Delhi NCT, India	Yes	0.52°	# Obs	44	79	82	76	74
South & West Asia			CH4:CO	1.04 (0.94, 1.13)	1.06 (1, 1.12)	1.05 (0.99, 1.11)	1.1 (1.04, 1.17)	1.25 (1.16, 1.34)
(28.65°, 77.24°)			CH4 [Tg]	0.91 (0.69, 1.15)	0.94 (0.7, 1.18)	0.94 (0.71, 1.18)	0.99 (0.76, 1.22)	1.13 (0.84, 1.43)
			EDGARv8 CH4 [Tg]	0.73	0.73	0.74	0.75	NA
			EDGARv2024 CH4 [Tg]	0.49	0.49	0.5	0.5	0.51
Detroit, USA	No	0.64°	# Obs	6	14	14	16	13
North America			CH4:CO	1.41 (0.96, 1.98)	0.87 (0.77, 0.98)	1.03 (0.91, 1.17)	1.09 (0.98, 1.2)	1.32 (0.98, 1.8)
(42.4°, -83.05°)			CH4 [Tg]	0.21 (0.13, 0.32)	0.12 (0.09, 0.15)	0.14 (0.11, 0.19)	0.16 (0.12, 0.2)	0.19 (0.13, 0.28)
			EDGARv8 CH4 [Tg]	0.27	0.27	0.27	0.28	NA
			EDGARv2024 CH4 [Tg]	0.26	0.27	0.27	0.27	0.28
Dhaka, Bangladesh	Yes	0.59°	# Obs	48	56	64	73	67
South & West Asia			CH4:CO	2.47 (2.16, 2.79)	2.43 (2.14, 2.74)	2.13 (1.89, 2.41)	1.87 (1.66, 2.1)	1.85 (1.67, 2.04)
(23.8°, 90.35°)			CH4 [Tg]	1.18 (0.88, 1.49)	1.15 (0.86, 1.47)	0.98 (0.73, 1.25)	0.88 (0.66, 1.12)	0.87 (0.65, 1.12)
			EDGARv8 CH4 [Tg]	0.95	0.96	0.97	0.98	NA
			EDGARv2024 CH4 [Tg]	0.72	0.74	0.75	0.76	0.78
Dubai, United Arab Emirates	Yes	0.72°	# Obs	0	0	7	5	20
South & West Asia			CH4:CO	NA	NA	1.22 (0.94, 1.59)	1.04 (0.92, 1.17)	1.54 (1.33, 1.77)
(25.34°, 55.4°)			CH4 [Tg]	NA	NA	0.48 (0.32, 0.68)	0.43 (0.32, 0.55)	0.64 (0.47, 0.82)
			EDGARv8 CH4 [Tg]	0.26	0.26	0.25	0.26	NA

Table S1. CH_4:CO and CH_4 Emissions Data *(continued)*

Name, Region, Location	C40	Box	Type	2019	2020	2021	2022	2023
			EDGARv2024 CH4 [Tg]	0.22	0.22	0.22	0.22	0.23
Durban (eThekwini), South Africa	Yes	0.66°	# Obs	18	30	32	37	38
Africa			CH4:CO	0.94 (0.82, 1.08)	1.12 (0.97, 1.3)	1.03 (0.94, 1.12)	1.11 (1.03, 1.19)	1.22 (1.11, 1.35)
(-29.75°, 31°)			CH4 [Tg]	0.19 (0.14, 0.24)	0.22 (0.16, 0.28)	0.2 (0.15, 0.24)	0.21 (0.16, 0.26)	0.23 (0.17, 0.29)
			EDGARv8 CH4 [Tg]	0.11	0.11	0.11	0.11	NA
			EDGARv2024 CH4 [Tg]	0.09	0.09	0.09	0.09	0.09
Freetown, Sierra Leone	Yes	0.66°	# Obs	5	0	0	0	6
Africa			CH4:CO	0.69 (0.59, 0.8)	NA	NA	NA	1.01 (0.75, 1.28)
(8.47°, -13.23°)			CH4 [Tg]	0.09 (0.06, 0.12)	NA	NA	NA	0.13 (0.09, 0.18)
			EDGARv8 CH4 [Tg]	0.06	0.06	0.06	0.06	NA
			EDGARv2024 CH4 [Tg]	0.06	0.06	0.06	0.06	0.06
Guadalajara, Mexico	Yes	0.48°	# Obs	21	25	24	28	23
Latin America			CH4:CO	1.2 (1.04, 1.39)	1.21 (1.08, 1.34)	1.31 (1.14, 1.52)	1.28 (1.12, 1.44)	1.5 (1.35, 1.67)
(20.63°, -103.28°)			CH4 [Tg]	0.1 (0.07, 0.13)	0.08 (0.06, 0.11)	0.1 (0.07, 0.12)	0.1 (0.08, 0.13)	0.12 (0.09, 0.15)
			EDGARv8 CH4 [Tg]	0.17	0.17	0.16	0.17	NA
			EDGARv2024 CH4 [Tg]	0.15	0.15	0.13	0.14	0.14
Guangzhou, China	Yes	0.49°	# Obs	36	25	29	31	29
Central East Asia			CH4:CO	1.16 (1.04, 1.3)	1.19 (1, 1.42)	1.04 (0.92, 1.2)	1.02 (0.87, 1.2)	1.01 (0.89, 1.19)
(23.15°, 113.28°)			CH4 [Tg]	0.53 (0.41, 0.68)	0.55 (0.4, 0.73)	0.49 (0.36, 0.63)	0.48 (0.35, 0.63)	0.48 (0.36, 0.62)
			EDGARv8 CH4 [Tg]	0.74	0.75	0.76	0.77	NA
			EDGARv2024 CH4 [Tg]	0.57	0.58	0.6	0.61	0.63
Hangzhou, China	Yes	0.54°	# Obs	32	23	31	21	32
Central East Asia			CH4:CO	0.82 (0.73, 0.92)	0.93 (0.75, 1.16)	0.87 (0.78, 0.97)	0.8 (0.72, 0.86)	0.9 (0.8, 1.01)
(30.26°, 120.19°)			CH4 [Tg]	0.31 (0.23, 0.39)	0.35 (0.25, 0.48)	0.33 (0.25, 0.42)	0.31 (0.23, 0.39)	0.35 (0.25, 0.44)
			EDGARv8 CH4 [Tg]	0.49	0.5	0.51	0.51	NA
			EDGARv2024 CH4 [Tg]	0.42	0.43	0.43	0.43	0.44
Hanoi, Vietnam	Yes	0.53°	# Obs	18	15	22	20	22
East, Southeast Asia & Oceania			CH4:CO	0.99 (0.83, 1.18)	0.88 (0.72, 1.1)	1.16 (1.01, 1.34)	1.01 (0.84, 1.2)	1 (0.83, 1.16)
(21.02°, 105.84°)			CH4 [Tg]	0.35 (0.26, 0.46)	0.31 (0.22, 0.43)	0.43 (0.31, 0.55)	0.4 (0.29, 0.52)	0.39 (0.28, 0.5)
			EDGARv8 CH4 [Tg]	0.49	0.46	0.46	0.5	NA
			EDGARv2024 CH4 [Tg]	0.43	0.4	0.41	0.45	0.46
Heidelberg, Germany	Yes	0.8°	# Obs	9	27	22	21	28
Europe			CH4:CO	1.43 (1.03, 1.98)	1.17 (1.04, 1.32)	1.18 (1.01, 1.41)	1.59 (1.24, 2)	1.19 (0.98, 1.44)
(49.48°, 8.48°)			CH4 [Tg]	0.22 (0.15, 0.33)	0.17 (0.13, 0.22)	0.19 (0.14, 0.24)	0.25 (0.17, 0.34)	0.18 (0.13, 0.24)
			EDGARv8 CH4 [Tg]	0.15	0.15	0.15	0.14	NA
			EDGARv2024 CH4 [Tg]	0.14	0.13	0.13	0.13	0.12
Houston,TX, USA	Yes	0.7°	# Obs	0	13	15	21	24
North America			CH4:CO	NA	1.21 (1.01, 1.42)	1.11 (0.99, 1.25)	1.19 (1.09, 1.32)	1.22 (1.05, 1.46)
(29.76°, -95.35°)			CH4 [Tg]	NA	0.17 (0.12, 0.22)	0.17 (0.13, 0.22)	0.19 (0.14, 0.24)	0.2 (0.14, 0.26)
			EDGARv8 CH4 [Tg]	0.15	0.15	0.15	0.15	NA
			EDGARv2024 CH4 [Tg]	0.15	0.15	0.15	0.15	0.16
Hyderabad, India	No	0.53°	# Obs	8	15	21	30	39
South & West Asia			CH4:CO	1.02 (0.82, 1.19)	1.07 (0.96, 1.18)	1.06 (0.94, 1.19)	1.05 (0.95, 1.15)	1.22 (1.12, 1.32)
(17.36°, 78.53°)			CH4 [Tg]	0.25 (0.18, 0.33)	0.26 (0.2, 0.33)	0.27 (0.2, 0.34)	0.27 (0.2, 0.34)	0.31 (0.23, 0.39)
			EDGARv8 CH4 [Tg]	0.41	0.51	0.49	0.49	NA
			EDGARv2024 CH4 [Tg]	0.35	0.45	0.43	0.42	0.43
Istanbul, Türkiye	Yes	0.62°	# Obs	5	9	6	14	28
Europe			CH4:CO	1.68 (1.06, 2.35)	1.1 (0.96, 1.26)	0.99 (0.84, 1.15)	1.11 (0.98, 1.23)	1.43 (1.22, 1.65)
(41.06°, 28.86°)			CH4 [Tg]	0.24 (0.14, 0.36)	0.17 (0.12, 0.22)	0.16 (0.12, 0.21)	0.19 (0.14, 0.24)	0.24 (0.17, 0.31)
			EDGARv8 CH4 [Tg]	0.27	0.28	0.29	0.3	NA
			EDGARv2024 CH4 [Tg]	0.23	0.24	0.25	0.26	0.27
Johannesburg, South Africa	Yes	0.57°	# Obs	44	36	45	38	45

Table S1. CH_4**:CO and** CH_4 **Emissions Data** ***(continued)***

Name, Region, Location	C40	Box	Type	2019	2020	2021	2022	2023
Africa			CH4:CO	0.82 (0.75, 0.9)	0.87 (0.8, 0.94)	0.92 (0.85, 0.98)	0.96 (0.88, 1.05)	1.04 (0.98, 1.11)
(-26.19°, 28.05°)			CH4 [Tg]	0.6 (0.46, 0.75)	0.6 (0.44, 0.75)	0.64 (0.47, 0.79)	0.64 (0.48, 0.8)	0.7 (0.54, 0.88)
			EDGARv8 CH4 [Tg]	0.63	0.64	0.66	0.63	NA
			EDGARv2024 CH4 [Tg]	0.54	0.55	0.57	0.54	0.54
Kansas City, USA	No	0.58°	# Obs	7	20	27	28	15
North America			CH4:CO	0.96 (0.78, 1.21)	0.87 (0.7, 1.08)	0.9 (0.8, 1.01)	0.9 (0.81, 1.02)	0.96 (0.85, 1.07)
(39.11°, -94.53°)			CH4 [Tg]	0.07 (0.05, 0.1)	0.06 (0.04, 0.08)	0.06 (0.05, 0.08)	0.07 (0.05, 0.08)	0.07 (0.05, 0.09)
			EDGARv8 CH4 [Tg]	0.07	0.07	0.07	0.07	NA
			EDGARv2024 CH4 [Tg]	0.06	0.06	0.06	0.06	0.06
Karachi, Pakistan	Yes	0.66°	# Obs	5	21	21	36	44
South & West Asia			CH4:CO	2.05 (1.66, 2.46)	1.58 (1.29, 1.9)	1.72 (1.41, 2.03)	1.71 (1.49, 1.95)	1.47 (1.31, 1.62)
(24.86°, 67.02°)			CH4 [Tg]	0.87 (0.6, 1.15)	0.68 (0.48, 0.9)	0.76 (0.55, 0.99)	0.75 (0.55, 0.95)	0.64 (0.48, 0.8)
			EDGARv8 CH4 [Tg]	0.51	0.53	0.54	0.55	NA
			EDGARv2024 CH4 [Tg]	0.31	0.34	0.35	0.35	0.35
Kolkata, India	Yes	0.66°	# Obs	58	75	71	79	82
South & West Asia			CH4:CO	0.86 (0.75, 1.01)	0.94 (0.85, 1.03)	0.93 (0.86, 1.02)	0.95 (0.89, 1.02)	1.03 (0.95, 1.11)
(22.49°, 88.3°)			CH4 [Tg]	0.53 (0.39, 0.7)	0.58 (0.44, 0.73)	0.59 (0.44, 0.75)	0.61 (0.46, 0.76)	0.65 (0.49, 0.82)
			EDGARv8 CH4 [Tg]	0.45	0.46	0.46	0.47	NA
			EDGARv2024 CH4 [Tg]	0.37	0.38	0.38	0.39	0.38
Kyiv, Ukraine	No	0.64°	# Obs	6	15	18	12	23
Europe			CH4:CO	1.08 (0.93, 1.27)	1.32 (1.06, 1.62)	1.34 (1.12, 1.58)	1.05 (0.88, 1.29)	1.54 (1.14, 2.26)
(50.4°, 30.63°)			CH4 [Tg]	0.08 (0.06, 0.1)	0.1 (0.07, 0.13)	0.09 (0.07, 0.12)	0.06 (0.04, 0.08)	0.09 (0.06, 0.14)
			EDGARv8 CH4 [Tg]	0.11	0.11	0.11	0.11	NA
			EDGARv2024 CH4 [Tg]	0.11	0.11	0.11	0.11	0.11
Lagos, Nigeria	Yes	0.69°	# Obs	5	0	0	5	5
Africa			CH4:CO	0.66 (0.57, 0.75)	NA	NA	0.94 (0.84, 1.06)	0.99 (0.84, 1.16)
(6.46°, 3.34°)			CH4 [Tg]	1.08 (0.78, 1.39)	NA	NA	1.6 (1.19, 2.03)	1.68 (1.24, 2.2)
			EDGARv8 CH4 [Tg]	0.99	1.02	1.04	1.05	NA
			EDGARv2024 CH4 [Tg]	0.74	0.76	0.78	0.79	0.79
Lahore, Pakistan	No	0.48°	# Obs	29	71	54	58	50
South & West Asia			CH4:CO	1.61 (1.45, 1.76)	1.52 (1.41, 1.63)	1.5 (1.41, 1.61)	1.41 (1.32, 1.5)	1.42 (1.33, 1.52)
(31.58°, 74.31°)			CH4 [Tg]	0.78 (0.58, 0.98)	0.74 (0.57, 0.94)	0.75 (0.56, 0.92)	0.7 (0.52, 0.88)	0.71 (0.53, 0.88)
			EDGARv8 CH4 [Tg]	0.66	0.68	0.7	0.71	NA
			EDGARv2024 CH4 [Tg]	0.52	0.55	0.57	0.57	0.6
Lisbon, Portugal	Yes	0.76°	# Obs	0	0	16	14	34
Europe			CH4:CO	NA	NA	1.11 (0.93, 1.31)	1.18 (1.01, 1.36)	1.07 (0.97, 1.17)
(38.75°, -9.28°)			CH4 [Tg]	NA	NA	0.04 (0.03, 0.06)	0.05 (0.03, 0.06)	0.04 (0.03, 0.05)
			EDGARv8 CH4 [Tg]	0.13	0.13	0.13	0.13	NA
			EDGARv2024 CH4 [Tg]	0.12	0.12	0.12	0.12	0.13
London, United Kingdom	Yes	0.58°	# Obs	9	11	10	25	27
Europe			CH4:CO	1.96 (1, 3.25)	1.18 (0.76, 1.69)	1.44 (0.98, 2.2)	1.51 (1.11, 2.1)	1.27 (1.01, 1.63)
(51.53°, -0.2°)			CH4 [Tg]	0.18 (0.08, 0.3)	0.1 (0.06, 0.15)	0.11 (0.07, 0.19)	0.12 (0.07, 0.17)	0.1 (0.07, 0.14)
			EDGARv8 CH4 [Tg]	0.13	0.13	0.13	0.13	NA
			EDGARv2024 CH4 [Tg]	0.12	0.12	0.12	0.12	0.11
Los Angeles,CA, USA	Yes	0.62°	# Obs	36	44	51	45	57
North America			CH4:CO	1.29 (1.13, 1.47)	1.17 (1.07, 1.28)	1.21 (1.12, 1.31)	1.33 (1.19, 1.47)	1.38 (1.3, 1.48)
(34.06°, -118.27°)			CH4 [Tg]	0.29 (0.21, 0.37)	0.24 (0.18, 0.29)	0.27 (0.2, 0.33)	0.3 (0.22, 0.38)	0.31 (0.23, 0.39)
			EDGARv8 CH4 [Tg]	0.5	0.54	0.5	0.5	NA
			EDGARv2024 CH4 [Tg]	0.5	0.54	0.5	0.5	0.5
Luanda, Angola	No	0.86°	# Obs	9	5	16	6	7
Africa			CH4:CO	1.11 (0.9, 1.37)	1.21 (0.93, 1.47)	1.07 (0.89, 1.29)	1.28 (1.01, 1.59)	1.47 (0.85, 2.37)
(-8.82°, 13.28°)			CH4 [Tg]	0.39 (0.28, 0.53)	0.43 (0.29, 0.57)	0.39 (0.29, 0.51)	0.47 (0.32, 0.64)	0.53 (0.29, 0.91)

Table S1. CH_4:CO and CH_4 Emissions Data *(continued)*

Name, Region, Location	C40	Box	Type	2019	2020	2021	2022	2023
			EDGARv8 CH4 [Tg]	0.18	0.19	0.19	0.2	NA
			EDGARv2024 CH4 [Tg]	0.13	0.13	0.14	0.14	0.14
Madrid, Spain	Yes	0.69°	# Obs	0	5	11	10	21
Europe			CH4:CO	NA	1.29 (1.02, 1.59)	1.45 (1.04, 1.88)	1.31 (1.16, 1.46)	1.29 (1.18, 1.41)
(40.44°, -3.7°)			CH4 [Tg]	NA	0.07 (0.05, 0.1)	0.08 (0.05, 0.11)	0.07 (0.06, 0.09)	0.07 (0.05, 0.09)
			EDGARv8 CH4 [Tg]	0.11	0.11	0.11	0.11	NA
			EDGARv2024 CH4 [Tg]	0.1	0.1	0.1	0.1	0.1
Manchester, United Kingdom	No	0.77°	# Obs	11	8	5	10	19
Europe			CH4:CO	1.67 (1.04, 2.5)	1.34 (0.76, 2.17)	1.13 (0.79, 1.66)	1.69 (1.31, 2.08)	1.34 (1.1, 1.65)
(53.45°, -2.23°)			CH4 [Tg]	0.16 (0.1, 0.26)	0.12 (0.06, 0.2)	0.1 (0.06, 0.15)	0.14 (0.1, 0.19)	0.11 (0.08, 0.15)
			EDGARv8 CH4 [Tg]	0.26	0.26	0.26	0.25	NA
			EDGARv2024 CH4 [Tg]	0.25	0.24	0.24	0.24	0.24
Melbourne, Australia	Yes	0.79°	# Obs	8	9	6	8	19
East, Southeast Asia & Oceania			CH4:CO	0.63 (0.33, 0.97)	1.1 (0.89, 1.38)	1.35 (0.87, 1.92)	1.27 (1.04, 1.51)	1.17 (1.04, 1.32)
(-37.82°, 144.96°)			CH4 [Tg]	0.07 (0.04, 0.11)	0.12 (0.08, 0.16)	0.15 (0.09, 0.22)	0.14 (0.1, 0.18)	0.13 (0.09, 0.17)
			EDGARv8 CH4 [Tg]	0.18	0.17	0.17	0.18	NA
			EDGARv2024 CH4 [Tg]	0.18	0.17	0.17	0.17	0.17
Mexico City, Mexico	Yes	0.57°	# Obs	21	11	16	14	10
Latin America			CH4:CO	1.07 (0.96, 1.18)	1.25 (1.04, 1.46)	1.12 (0.92, 1.37)	1.1 (0.9, 1.35)	1.09 (0.89, 1.3)
(19.38°, -99.21°)			CH4 [Tg]	0.27 (0.2, 0.34)	0.27 (0.2, 0.35)	0.25 (0.18, 0.33)	0.26 (0.19, 0.35)	0.26 (0.18, 0.35)
			EDGARv8 CH4 [Tg]	0.23	0.24	0.24	0.24	NA
			EDGARv2024 CH4 [Tg]	0.14	0.14	0.14	0.14	0.14
Miami,FL, USA	Yes	0.69°	# Obs	0	0	0	5	10
North America			CH4:CO	NA	NA	NA	1.32 (0.86, 1.98)	1.16 (0.91, 1.4)
(25.77°, -80.22°)			CH4 [Tg]	NA	NA	NA	0.11 (0.07, 0.17)	0.1 (0.07, 0.13)
			EDGARv8 CH4 [Tg]	0.07	0.07	0.07	0.07	NA
			EDGARv2024 CH4 [Tg]	0.07	0.07	0.07	0.07	0.07
Milan, Italy	Yes	0.63°	# Obs	7	23	33	25	41
Europe			CH4:CO	1.7 (1.01, 2.72)	1.39 (1.18, 1.62)	1.43 (1.25, 1.63)	1.32 (1.17, 1.49)	1.26 (1.13, 1.4)
(45.47°, 9.14°)			CH4 [Tg]	0.28 (0.15, 0.47)	0.21 (0.15, 0.27)	0.22 (0.17, 0.28)	0.2 (0.15, 0.26)	0.19 (0.14, 0.24)
			EDGARv8 CH4 [Tg]	0.33	0.32	0.33	0.32	NA
			EDGARv2024 CH4 [Tg]	0.31	0.31	0.31	0.3	0.3
Monterrey, Mexico	No	0.58°	# Obs	19	27	22	35	34
Latin America			CH4:CO	1.07 (0.94, 1.2)	1.29 (1.15, 1.41)	1.32 (1.18, 1.48)	1.14 (1.01, 1.27)	1.06 (0.95, 1.18)
(25.69°, -100.42°)			CH4 [Tg]	0.12 (0.09, 0.16)	0.13 (0.09, 0.16)	0.13 (0.1, 0.17)	0.13 (0.1, 0.16)	0.12 (0.09, 0.15)
			EDGARv8 CH4 [Tg]	0.16	0.16	0.16	0.17	NA
			EDGARv2024 CH4 [Tg]	0.08	0.08	0.08	0.08	0.08
Montréal, Canada	Yes	0.57°	# Obs	17	8	21	25	27
North America			CH4:CO	1.14 (0.99, 1.33)	1.48 (1.05, 1.97)	1.05 (0.91, 1.24)	1.05 (0.9, 1.21)	1.31 (1.15, 1.47)
(45.5°, -73.58°)			CH4 [Tg]	0.13 (0.1, 0.17)	0.17 (0.11, 0.23)	0.12 (0.09, 0.16)	0.12 (0.09, 0.15)	0.15 (0.11, 0.19)
			EDGARv8 CH4 [Tg]	0.16	0.17	0.17	0.17	NA
			EDGARv2024 CH4 [Tg]	0.16	0.16	0.16	0.17	0.17
Moscow, Russia	No	0.56°	# Obs	14	13	38	31	25
Europe			CH4:CO	1.23 (0.99, 1.5)	1.19 (0.82, 1.7)	1.03 (0.91, 1.19)	1.31 (1.12, 1.52)	1.18 (1, 1.41)
(55.82°, 37.76°)			CH4 [Tg]	0.45 (0.32, 0.59)	0.41 (0.26, 0.63)	0.39 (0.29, 0.51)	0.5 (0.37, 0.65)	0.45 (0.33, 0.59)
			EDGARv8 CH4 [Tg]	0.48	0.49	0.51	0.51	NA
			EDGARv2024 CH4 [Tg]	0.46	0.48	0.49	0.51	0.53
Mumbai, India	Yes	0.86°	# Obs	39	42	42	60	56
South & West Asia			CH4:CO	1.05 (0.96, 1.16)	1.18 (1.06, 1.31)	1.27 (1.13, 1.44)	1.16 (1.07, 1.27)	1.16 (1.07, 1.26)
(18.97°, 72.81°)			CH4 [Tg]	1.05 (0.8, 1.34)	1.15 (0.86, 1.46)	1.31 (0.98, 1.67)	1.22 (0.93, 1.52)	1.21 (0.9, 1.54)
			EDGARv8 CH4 [Tg]	0.64	0.63	0.65	0.67	NA
			EDGARv2024 CH4 [Tg]	0.4	0.39	0.4	0.41	0.39

Table S1. CH_4:CO and CH_4 Emissions Data *(continued)*

Name, Region, Location	C40	Box	Type	2019	2020	2021	2022	2023
Nanjing, China	Yes	0.53°	# Obs	46	35	58	44	61
Central East Asia			CH4:CO	0.52 (0.47, 0.6)	0.63 (0.55, 0.7)	0.52 (0.48, 0.57)	0.56 (0.51, 0.63)	0.7 (0.6, 0.82)
(32.03°, 118.75°)			CH4 [Tg]	0.49 (0.36, 0.63)	0.57 (0.42, 0.73)	0.5 (0.38, 0.62)	0.54 (0.41, 0.68)	0.68 (0.49, 0.89)
			EDGARv8 CH4 [Tg]	0.55	0.55	0.56	0.56	NA
			EDGARv2024 CH4 [Tg]	0.46	0.47	0.47	0.47	0.48
New Orleans,LA, USA	Yes	0.5°	# Obs	6	17	16	28	20
North America			CH4:CO	1.1 (0.75, 1.5)	1.1 (0.94, 1.25)	1.21 (0.99, 1.45)	1.25 (1.13, 1.4)	1.46 (1.16, 1.9)
(29.96°, -90.1°)			CH4 [Tg]	0.05 (0.03, 0.07)	0.05 (0.03, 0.06)	0.06 (0.04, 0.08)	0.06 (0.05, 0.08)	0.07 (0.05, 0.1)
			EDGARv8 CH4 [Tg]	0.04	0.04	0.04	0.04	NA
			EDGARv2024 CH4 [Tg]	0.04	0.04	0.04	0.04	0.04
New York City,NY, USA	Yes	0.64°	# Obs	11	14	14	32	40
North America			CH4:CO	1.36 (1.01, 1.78)	1.15 (0.93, 1.39)	1.14 (0.91, 1.39)	1.3 (1.07, 1.66)	1.17 (1.05, 1.3)
(40.77°, -73.95°)			CH4 [Tg]	0.46 (0.31, 0.63)	0.34 (0.25, 0.46)	0.38 (0.27, 0.51)	0.44 (0.31, 0.6)	0.4 (0.3, 0.5)
			EDGARv8 CH4 [Tg]	0.17	0.17	0.17	0.18	NA
			EDGARv2024 CH4 [Tg]	0.17	0.17	0.17	0.17	0.17
Oslo, Norway	Yes	0.57°	# Obs	12	12	12	14	20
Europe			CH4:CO	1.32 (0.97, 1.69)	1.14 (0.94, 1.41)	0.98 (0.84, 1.13)	1.34 (1.1, 1.62)	1.14 (0.93, 1.36)
(59.92°, 10.74°)			CH4 [Tg]	0.03 (0.02, 0.04)	0.03 (0.02, 0.04)	0.02 (0.02, 0.03)	0.03 (0.02, 0.04)	0.03 (0.02, 0.04)
			EDGARv8 CH4 [Tg]	0.03	0.04	0.04	0.04	NA
			EDGARv2024 CH4 [Tg]	0.03	0.03	0.03	0.03	0.03
Paris, France	Yes	0.58°	# Obs	9	7	10	13	26
Europe			CH4:CO	1.63 (0.8, 2.66)	1.1 (0.68, 1.58)	1.37 (0.97, 1.95)	1.37 (1.11, 1.64)	1.2 (0.97, 1.52)
(48.9°, 2.34°)			CH4 [Tg]	0.31 (0.14, 0.52)	0.19 (0.11, 0.29)	0.26 (0.17, 0.39)	0.25 (0.18, 0.34)	0.22 (0.16, 0.3)
			EDGARv8 CH4 [Tg]	0.17	0.16	0.16	0.16	NA
			EDGARv2024 CH4 [Tg]	0.14	0.13	0.13	0.13	0.12
Philadelphia,PA, USA	Yes	0.56°	# Obs	10	12	22	27	36
North America			CH4:CO	1.01 (0.79, 1.29)	1.3 (0.98, 1.66)	1.12 (0.94, 1.32)	1.1 (0.98, 1.24)	1.21 (1.06, 1.4)
(39.92°, -75.16°)			CH4 [Tg]	0.17 (0.12, 0.23)	0.19 (0.13, 0.26)	0.18 (0.13, 0.24)	0.18 (0.14, 0.23)	0.2 (0.15, 0.26)
			EDGARv8 CH4 [Tg]	0.15	0.15	0.15	0.15	NA
			EDGARv2024 CH4 [Tg]	0.15	0.15	0.15	0.15	0.15
Phoenix,AZ, USA	Yes	0.58°	# Obs	0	6	13	32	19
North America			CH4:CO	NA	1.13 (0.85, 1.54)	1.33 (1.05, 1.67)	1.14 (0.96, 1.32)	1.14 (0.92, 1.32)
(33.41°, -111.93°)			CH4 [Tg]	NA	0.09 (0.06, 0.13)	0.11 (0.08, 0.15)	0.1 (0.07, 0.12)	0.1 (0.07, 0.12)
			EDGARv8 CH4 [Tg]	0.25	0.27	0.22	0.26	NA
			EDGARv2024 CH4 [Tg]	0.24	0.27	0.22	0.26	0.26
Portland,OR, USA	Yes	0.57°	# Obs	12	25	28	38	42
North America			CH4:CO	1.29 (0.97, 1.79)	1.01 (0.86, 1.23)	1.08 (0.91, 1.26)	0.96 (0.86, 1.06)	1.08 (0.94, 1.22)
(45.51°, -122.69°)			CH4 [Tg]	0.1 (0.07, 0.15)	0.07 (0.05, 0.09)	0.08 (0.06, 0.11)	0.08 (0.06, 0.09)	0.08 (0.06, 0.11)
			EDGARv8 CH4 [Tg]	0.03	0.03	0.03	0.03	NA
			EDGARv2024 CH4 [Tg]	0.03	0.03	0.03	0.03	0.03
Qingdao, China	Yes	0.46°	# Obs	28	14	23	23	42
Central East Asia			CH4:CO	0.61 (0.52, 0.7)	0.62 (0.52, 0.72)	0.59 (0.5, 0.7)	0.73 (0.61, 0.86)	0.86 (0.74, 0.98)
(36.1°, 120.37°)			CH4 [Tg]	0.11 (0.08, 0.14)	0.11 (0.08, 0.14)	0.11 (0.08, 0.15)	0.14 (0.1, 0.18)	0.16 (0.12, 0.2)
			EDGARv8 CH4 [Tg]	0.19	0.19	0.2	0.2	NA
			EDGARv2024 CH4 [Tg]	0.14	0.14	0.14	0.15	0.15
Rio de Janeiro, Brazil	Yes	0.69°	# Obs	7	0	6	6	28
Latin America			CH4:CO	0.96 (0.74, 1.18)	NA	1.23 (0.81, 1.74)	1.14 (0.92, 1.42)	1.43 (1.23, 1.7)
(-22.99°, -43.25°)			CH4 [Tg]	0.29 (0.2, 0.39)	NA	0.39 (0.24, 0.58)	0.35 (0.25, 0.47)	0.43 (0.32, 0.56)
			EDGARv8 CH4 [Tg]	0.25	0.25	0.26	0.27	NA
			EDGARv2024 CH4 [Tg]	0.2	0.2	0.2	0.22	0.21
Rome, Italy	Yes	0.68°	# Obs	5	20	15	19	21
Europe			CH4:CO	1.03 (0.77, 1.42)	1.37 (1.15, 1.62)	1.14 (0.97, 1.34)	1.32 (1.03, 1.65)	1.27 (1.05, 1.48)

Table S1. CH_4:CO and CH_4 Emissions Data *(continued)*

Name, Region, Location	C40	Box	Type	2019	2020	2021	2022	2023
(41.87°, 12.53°)			CH4 [Tg]	0.16 (0.11, 0.23)	0.19 (0.14, 0.25)	0.16 (0.12, 0.22)	0.19 (0.13, 0.26)	0.18 (0.13, 0.24)
			EDGARv8 CH4 [Tg]	0.07	0.06	0.06	0.06	NA
			EDGARv2024 CH4 [Tg]	0.07	0.06	0.06	0.06	0.06
Salvador, Brazil	Yes	0.69°	# Obs	0	0	0	0	5
Latin America			CH4:CO	NA	NA	NA	NA	1.27 (0.96, 1.61)
(-13°, -38.48°)			CH4 [Tg]	NA	NA	NA	NA	0.11 (0.08, 0.16)
			EDGARv8 CH4 [Tg]	0.21	0.22	0.22	0.2	NA
			EDGARv2024 CH4 [Tg]	0.16	0.16	0.16	0.16	0.16
San Francisco,CA, USA	Yes	0.6°	# Obs	7	13	11	17	42
North America			CH4:CO	1.18 (0.95, 1.41)	1.01 (0.82, 1.22)	1.12 (0.82, 1.45)	1.29 (1.05, 1.67)	1.43 (1.25, 1.63)
(37.79°, -122.41°)			CH4 [Tg]	0.15 (0.11, 0.2)	0.12 (0.08, 0.16)	0.15 (0.1, 0.21)	0.17 (0.12, 0.25)	0.19 (0.14, 0.24)
			EDGARv8 CH4 [Tg]	0.15	0.16	0.15	0.15	NA
			EDGARv2024 CH4 [Tg]	0.14	0.15	0.15	0.15	0.15
Santiago, Chile	Yes	0.51°	# Obs	0	0	0	0	6
Latin America			CH4:CO	NA	NA	NA	NA	1.16 (0.97, 1.4)
(-33.5°, -70.76°)			CH4 [Tg]	NA	NA	NA	NA	0.16 (0.12, 0.22)
			EDGARv8 CH4 [Tg]	0.32	0.31	0.3	0.32	NA
			EDGARv2024 CH4 [Tg]	0.27	0.25	0.23	0.24	0.24
Seattle,WA, USA	Yes	0.83°	# Obs	0	0	0	0	10
North America			CH4:CO	NA	NA	NA	NA	1.19 (1, 1.44)
(47.61°, -122.33°)			CH4 [Tg]	NA	NA	NA	NA	0.14 (0.1, 0.19)
			EDGARv8 CH4 [Tg]	0.08	0.08	0.08	0.08	NA
			EDGARv2024 CH4 [Tg]	0.08	0.08	0.08	0.08	0.08
Seoul, South Korea	Yes	0.75°	# Obs	40	35	27	36	35
East, Southeast Asia & Oceania			CH4:CO	0.84 (0.75, 0.93)	0.92 (0.83, 1.01)	1.04 (0.92, 1.17)	0.98 (0.88, 1.09)	1.03 (0.93, 1.15)
(37.49°, 126.95°)			CH4 [Tg]	0.81 (0.6, 1.03)	0.83 (0.63, 1.06)	1 (0.74, 1.26)	0.88 (0.66, 1.12)	0.94 (0.7, 1.2)
			EDGARv8 CH4 [Tg]	0.77	0.84	0.87	0.88	NA
			EDGARv2024 CH4 [Tg]	0.64	0.69	0.75	0.76	0.75
Shanghai, China	Yes	0.76°	# Obs	28	19	31	22	45
Central East Asia			CH4:CO	0.52 (0.45, 0.59)	0.61 (0.51, 0.71)	0.54 (0.48, 0.6)	0.6 (0.53, 0.67)	0.66 (0.59, 0.74)
(31.26°, 121.43°)			CH4 [Tg]	1.21 (0.89, 1.56)	1.41 (1.01, 1.83)	1.24 (0.92, 1.56)	1.39 (1.03, 1.76)	1.54 (1.15, 1.94)
			EDGARv8 CH4 [Tg]	1.42	1.44	1.46	1.47	NA
			EDGARv2024 CH4 [Tg]	1.15	1.17	1.19	1.2	1.23
Stockholm, Sweden	Yes	0.8°	# Obs	8	14	13	13	27
Europe			CH4:CO	0.93 (0.6, 1.33)	0.99 (0.85, 1.17)	1.06 (0.88, 1.25)	1.17 (0.95, 1.64)	1.04 (0.94, 1.16)
(59.31°, 18.08°)			CH4 [Tg]	0.03 (0.02, 0.05)	0.03 (0.03, 0.05)	0.04 (0.03, 0.05)	0.04 (0.03, 0.06)	0.04 (0.03, 0.05)
			EDGARv8 CH4 [Tg]	0.08	0.08	0.08	0.08	NA
			EDGARv2024 CH4 [Tg]	0.05	0.05	0.05	0.05	0.05
Sydney, Australia	Yes	0.69°	# Obs	6	10	7	5	20
East, Southeast Asia & Oceania			CH4:CO	1.27 (0.72, 1.96)	1.09 (0.93, 1.29)	1.23 (0.87, 1.64)	1.37 (0.96, 1.93)	1.35 (1.18, 1.56)
(-33.88°, 151.2°)			CH4 [Tg]	0.19 (0.11, 0.31)	0.16 (0.12, 0.21)	0.19 (0.12, 0.27)	0.21 (0.13, 0.32)	0.21 (0.15, 0.27)
			EDGARv8 CH4 [Tg]	0.18	0.17	0.16	0.16	NA
			EDGARv2024 CH4 [Tg]	0.21	0.19	0.18	0.17	0.17
São Paulo, Brazil	Yes	0.74°	# Obs	32	40	26	27	55
Latin America			CH4:CO	1.15 (1.03, 1.3)	1.11 (1.03, 1.2)	1.17 (1.08, 1.26)	1.22 (1.1, 1.35)	1.2 (1.13, 1.26)
(-23.54°, -46.65°)			CH4 [Tg]	0.55 (0.41, 0.7)	0.53 (0.39, 0.67)	0.57 (0.42, 0.71)	0.59 (0.43, 0.75)	0.57 (0.43, 0.71)
			EDGARv8 CH4 [Tg]	0.89	0.9	0.91	0.91	NA
			EDGARv2024 CH4 [Tg]	0.7	0.71	0.71	0.74	0.73
Tampa, USA	No	0.79°	# Obs	5	6	8	17	20
North America			CH4:CO	1.28 (0.97, 1.76)	1.19 (0.88, 1.79)	1.57 (0.96, 2.51)	1.28 (1.08, 1.51)	1.13 (0.98, 1.34)
(28.08°, -82.41°)			CH4 [Tg]	0.17 (0.12, 0.24)	0.14 (0.09, 0.23)	0.21 (0.12, 0.34)	0.18 (0.13, 0.23)	0.15 (0.11, 0.2)
			EDGARv8 CH4 [Tg]	0.07	0.07	0.07	0.07	NA

Table S1. CH_4:CO and CH_4 Emissions Data *(continued)*

Name, Region, Location	C40	Box	Type	2019	2020	2021	2022	2023
			EDGARv2024 CH4 [Tg]	0.06	0.06	0.06	0.06	0.06
Tehran, Iran	No	0.63°	# Obs	38	39	37	28	33
South & West Asia			CH4:CO	1.63 (1.51, 1.77)	1.61 (1.46, 1.75)	1.55 (1.43, 1.69)	1.67 (1.51, 1.84)	1.52 (1.4, 1.65)
(35.56°, 51.16°)			CH4 [Tg]	0.15 (0.11, 0.18)	0.14 (0.1, 0.18)	0.14 (0.1, 0.18)	0.16 (0.12, 0.2)	0.14 (0.11, 0.18)
			EDGARv8 CH4 [Tg]	0.27	0.28	0.28	0.28	NA
			EDGARv2024 CH4 [Tg]	0.19	0.2	0.2	0.21	0.21
Tel Aviv - Yafo, Israel	Yes	0.58°	# Obs	0	5	13	18	28
Europe			CH4:CO	NA	2.03 (1.25, 2.65)	1.29 (1.12, 1.51)	1.79 (1.42, 2.18)	1.56 (1.35, 1.81)
(31.9°, 35°)			CH4 [Tg]	NA	0.1 (0.05, 0.14)	0.06 (0.05, 0.08)	0.09 (0.07, 0.13)	0.08 (0.06, 0.11)
			EDGARv8 CH4 [Tg]	0.33	0.38	0.42	0.23	NA
			EDGARv2024 CH4 [Tg]	0.19	0.19	0.2	0.2	0.2
Tianjin, China	No	0.56°	# Obs	66	44	48	49	61
Central East Asia			CH4:CO	0.61 (0.55, 0.68)	0.59 (0.52, 0.67)	0.76 (0.67, 0.86)	0.74 (0.67, 0.82)	0.83 (0.7, 1.01)
(39.14°, 117.13°)			CH4 [Tg]	0.38 (0.29, 0.48)	0.37 (0.27, 0.47)	0.46 (0.33, 0.58)	0.45 (0.33, 0.57)	0.51 (0.37, 0.67)
			EDGARv8 CH4 [Tg]	0.44	0.45	0.46	0.46	NA
			EDGARv2024 CH4 [Tg]	0.33	0.34	0.35	0.36	0.37
Tijuana, Mexico	No	0.53°	# Obs	21	30	29	31	33
Latin America			CH4:CO	1.37 (1.1, 1.7)	1.22 (1.07, 1.37)	1.21 (1.06, 1.37)	1.42 (1.24, 1.64)	1.51 (1.34, 1.69)
(32.5°, -116.85°)			CH4 [Tg]	0.11 (0.08, 0.15)	0.08 (0.06, 0.11)	0.09 (0.07, 0.11)	0.11 (0.08, 0.14)	0.12 (0.09, 0.15)
			EDGARv8 CH4 [Tg]	0.16	0.2	0.17	0.16	NA
			EDGARv2024 CH4 [Tg]	0.14	0.18	0.15	0.14	0.14
Toronto, Canada	Yes	0.86°	# Obs	7	14	13	17	27
North America			CH4:CO	0.94 (0.72, 1.21)	1.05 (0.85, 1.37)	1.03 (0.88, 1.21)	1.13 (0.94, 1.32)	1.11 (0.94, 1.28)
(43.78°, -79.41°)			CH4 [Tg]	0.25 (0.18, 0.35)	0.26 (0.18, 0.36)	0.26 (0.19, 0.35)	0.29 (0.21, 0.38)	0.28 (0.2, 0.37)
			EDGARv8 CH4 [Tg]	0.29	0.29	0.3	0.31	NA
			EDGARv2024 CH4 [Tg]	0.27	0.28	0.28	0.29	0.29
Warsaw, Poland	Yes	0.57°	# Obs	9	11	17	12	24
Europe			CH4:CO	1.22 (0.9, 1.55)	0.93 (0.71, 1.17)	1.27 (1.07, 1.48)	1.37 (0.93, 1.96)	1.1 (0.91, 1.31)
(52.28°, 20.95°)			CH4 [Tg]	0.17 (0.11, 0.24)	0.13 (0.08, 0.17)	0.18 (0.13, 0.24)	0.19 (0.12, 0.29)	0.15 (0.11, 0.2)
			EDGARv8 CH4 [Tg]	0.1	0.1	0.1	0.1	NA
			EDGARv2024 CH4 [Tg]	0.1	0.1	0.1	0.1	0.1
Washington DC, USA	Yes	0.79°	# Obs	8	14	22	21	43
North America			CH4:CO	1 (0.81, 1.23)	1.15 (0.93, 1.42)	1.09 (0.92, 1.28)	1.1 (0.96, 1.27)	1.12 (1.02, 1.23)
(38.93°, -77.04°)			CH4 [Tg]	0.23 (0.16, 0.31)	0.24 (0.16, 0.32)	0.25 (0.18, 0.32)	0.25 (0.19, 0.33)	0.26 (0.2, 0.32)
			EDGARv8 CH4 [Tg]	0.13	0.13	0.13	0.13	NA
			EDGARv2024 CH4 [Tg]	0.12	0.12	0.12	0.12	0.12
Wuhan, China	Yes	0.36°	# Obs	38	20	43	28	29
Central East Asia			CH4:CO	0.7 (0.61, 0.8)	0.72 (0.6, 0.86)	0.7 (0.62, 0.81)	0.66 (0.57, 0.76)	0.73 (0.63, 0.84)
(30.58°, 114.25°)			CH4 [Tg]	0.51 (0.38, 0.66)	0.52 (0.38, 0.69)	0.52 (0.39, 0.67)	0.5 (0.37, 0.64)	0.55 (0.41, 0.71)
			EDGARv8 CH4 [Tg]	0.31	0.31	0.31	0.32	NA
			EDGARv2024 CH4 [Tg]	0.26	0.26	0.27	0.27	0.27
Xi'An, China	No	0.52°	# Obs	14	14	15	22	24
Central East Asia			CH4:CO	0.96 (0.87, 1.08)	0.85 (0.75, 1)	0.9 (0.75, 1.05)	0.89 (0.76, 1.02)	1.07 (0.93, 1.24)
(34.25°, 108.95°)			CH4 [Tg]	0.35 (0.27, 0.44)	0.31 (0.23, 0.4)	0.33 (0.24, 0.43)	0.33 (0.24, 0.42)	0.4 (0.29, 0.51)
			EDGARv8 CH4 [Tg]	0.23	0.23	0.23	0.24	NA
			EDGARv2024 CH4 [Tg]	0.17	0.17	0.17	0.18	0.18
Yokohama, Japan	Yes	0.8°	# Obs	19	20	30	18	41
East, Southeast Asia & Oceania			CH4:CO	0.77 (0.64, 0.92)	0.89 (0.74, 1.06)	0.91 (0.82, 1)	0.96 (0.87, 1.06)	1.05 (0.95, 1.14)
(35.62°, 139.73°)			CH4 [Tg]	0.7 (0.51, 0.9)	0.71 (0.51, 0.93)	0.8 (0.6, 1.03)	0.81 (0.61, 1.03)	0.89 (0.67, 1.11)
			EDGARv8 CH4 [Tg]	0.26	0.26	0.26	0.26	NA
			EDGARv2024 CH4 [Tg]	0.25	0.25	0.25	0.25	0.24

Table S2. Total summed CH_4 emissions in Tg/year shown for each year of study. The number of cities corresponds to the total number of cities observed for that year, contributing to the column total immediately to the right. Despite variations in the total list of cities observed annually, 72 cities appear consistently in the data every year and the summed emissions for this subset is located in fourth column. The total summed CH_4 emissions for 51 C40 and 21 non-C40 cities are shown in the fifth and sixth columns, respectively. The 95% Confidence Intervals, shown in brackets, represent absolute uncertainty for the observed CH_4 emissions across all cities (third column) and the uncertainty on the detection of change for the observed CH_4 emissions of the 72 consistent cities, 51 C40 cities, and 21 non-C40 cities (fourth through sixth column). The two methods for uncertainty analysis are described in the Methods and in SI S4.

Year	# Cities	Observed CH_4 Emissions [Tg/y]	Observed CH_4 Emissions [Tg/y] for 72 consistent cities	Observed CH_4 Emissions [Tg/y] for 51 C40 cities	Observed CH_4 Emissions [Tg/y] for 21 non-C40 cities
2019	76	25.9 [17.8, 33.3]	23.9 [22.9, 24.9]	18.5 [17.5, 19.5]	5.4 [5.0, 5.9]
2020	79	23.6 [16.6, 30.5]	23.0 [22.0, 24.0]	18.0 [17.1, 19.0]	5.0 [4.6, 5.4]
2021	83	25.6 [18.1, 33.2]	23.8 [22.8, 24.8]	18.6 [17.8, 19.5]	5.2 [4.8, 5.6]
2022	85	28.3 [20.1, 36.6]	24.7 [23.6, 25.7]	19.2 [18.2, 20.1]	5.5 [5.0, 5.9]
2023	92	31.2 [22.3, 40.4]	25.3 [24.3, 26.4]	19.8 [18.8, 20.8]	5.6 [5.1, 6.1]

2. Urban Domain Selection

As stated in the main text, in reference to the C40 network, we will refer to the metropolitan areas as the cities that are at the center, though surrounding urban areas will also be included. The C40 cities are likely strong contributors to the emissions patterns, due to having significant population and inventoried emissions in the urban core region. Figure S1 shows the population reported by the C40 cities compared to the population within the urban domains used in this study (1). This mean ratio between the C40 population and our larger urban domain population is 37%, and shows regional groupings depending on the population density of urban areas. Having a domain larger than the city proper may touch upon city emissions outside Scope 1 emissions, as waste is often transported outside of city boundaries to landfills.

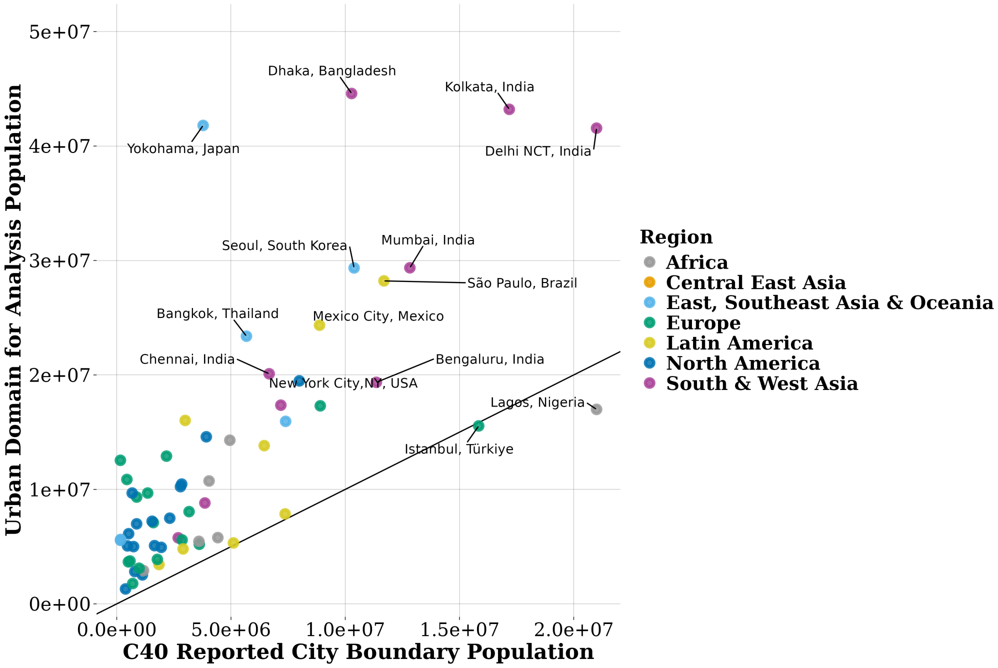

Fig. S1. Population differences reported by C40 cities and "city" domains used in analysis. Here we use the municipality names to represent the greater metropolitan areas that are incorporated in our larger urban domains. The solid black line is the one-to-one line between the C40 city population (1) and the population in our urban domain. C40 cities in Central East Asia did not report populations.

Some cities are very closely located and we could not disentangle emissions from each when urban domains overlapped. Closely neighboring cities are combined into one urban domain to avoid double counting of emissions, resulting in 90 C40 cities, rather than 96, to be evaluated. The combined urban domains are as follows: Amsterdam and Rotterdam reported as Amsterdam; Ekurhuleni, Johannesburg, and Tshwane are reported as Johannesburg; Tokyo and Yokohama are reported as Yokohama; and Nanjing and Zhenjiang are reported as Nanjing. Cities that did not have enough data pass filtering are not shown, including the Hong Kong and Shenzhen metropolitan area, which would have been combined if the area passed the filtering thresholds.

Plant et al., 2022 showed that as long as the city is well captured by the urban domain, the annual enhancement ratios are insensitive to varying domain size. If the domain is too small, not enough of the city and city emissions are captured to

evaluate the city as a point source. If the domain is too large, there is a higher chance of observing emissions from neighboring cities or other sources, yet attributing them to the city of interest. The domain boundaries are selected by mapping how population density of the selected area changes as the boundaries are expanded. We select a slope threshold of -0.45, where the total population density is decreasing as the area is growing. This threshold was set using sensitivity testing and examining the resulting domains by eye. The value for box size reported in Table S1 represents the distance from the center of the box to the edge latitude-wise and can therefore be thought of as the domain half-width. The domain is always square and the longitude-wise half width equals that of the latitude with an adjustment of $1/\cos(latitude)$. In Figure S2, we evaluate changing the domain size by nearly 50% and show limited sensitivity to the urban domain size, except on the lower extremity. We additionally find limited sensitivity when the center of the urban domain is shifted in any cardinal direction less than a quarter of a degree.

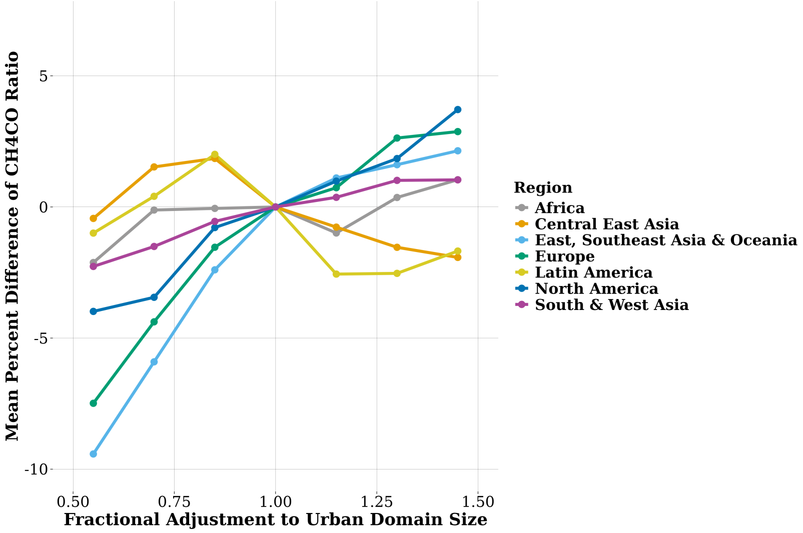

Fig. S2. Sensitivity study evaluating the impact of changing urban domain size. The regionally averaged percent difference in the annual average enhancement ratio for each region is shown with fractional adjustments made to the domain size. The domain box is still centered upon the city center peak in population density used in the main analysis. The domain size is changing significantly (nearly 50%, yet the annual enhancement ratio change is less than 10%.

Sectors typically thought of as urban sources of CH_4 emissions, including waste and emissions relating to natural gas usage, dominate the regional breakdown of sectoral EDGAR CH_4 emissions over urban domains in this study. Numerous studies, however, have shown these sectoral breakdowns are both much too low and missing sources, especially related to natural gas distribution and end-use (2–26). The sectoral breakdown of CH_4 emissions reported in EDGAR v8 and v2024 within our domains is shown in Figure S3. Reported emissions from agriculture account for 24% (EDGAR v8) to 28% (EDGAR v2024) of the total reported domain emissions. It is possible that the fraction of CH_4 emissions attributed to agriculture in the inventory may be too large. For example, Calgary in Canada has the second largest fraction of inventoried agricultural emissions in this study (80%). Xing et al., 2024 used TROPOMI with a mass balance approach to observe urban CH_4 emissions over Calgary where the urban domain is defined as the city's municipal boundaries. The area defined is much smaller than the urban domain we define around Calgary. However, the mean CH_4 emission rates are comparable across studies over the same time period. Xing et al., 2024 reports the mean emission rate estimate to be 0.079 ± 0.048 Tg/y, which includes our mean emission rate for the city of 0.033 Tg/y on average between 2020 and 2022. As stated earlier and in Plant et al., 2022, the enhancement ratio is not overly sensitive to changing the urban domain size, even though it may lead to different sectoral contributions reported in the CH_4 inventory.

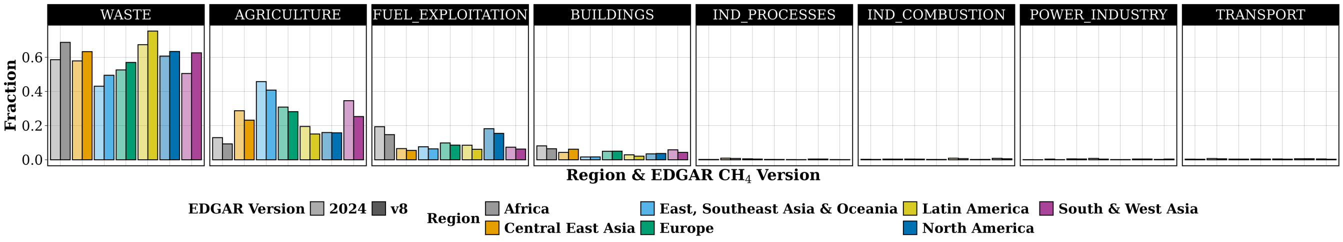

Fig. S3. The fraction by sector of bottom-up EDGAR CH_4 emissions in the urban domains for a region. The lighter shading indicates emissions from EDGAR version 2024 for the year 2022 while the darker shading for each region indicates CH_4 emissions from EDGAR version 8 for year 2022.

3. TROPOMI Data

TROPOMI data with Processor Version 02.04.00 or greater is used in this work. Orbits where there is no VIIRS-based cloud cover data are disregarded (27). The TROPOMI team produces a CO corrected product, but it is not used here as there are known striping issues with both the CO and CH_4. Using one "destriped" product with a non "destriped" product could increase bias due to the use of a ratio in this method. Table S3 outlines the filtering requirements applied to the TROPOMI CH_4 and CO data at the pixel level. Table S4 outlines the filtering thresholds performed at the urban domain level before calculating the daily enhancement ratios. The amount of data that meets filtering thresholds is listed by region in Table S5. One change made from the Plant et al., 2022 to this work is the urban domain selection method, in which we apply a box based on population density. This results in our urban domain being larger than what was used in Plant et al., 2022 for US cities. As a result, we also adjust the filtering threshold for data coverage to be 20% of the area of the domain must be covered by co-located TROPOMI CH_4 and CO pixels that pass meet all previous filtering requirements. Only co-located pixels are used in calculation of the summed enhancement ratio, so this filtering step is applied after all filtering listed in Table S3. A sensitivity study revealed the regionally averaged enhancement ratios were insensitive to area coverage thresholds between 10 to 20% and that by eye, overpasses looked well filled especially over the dense urban core regions with a minimum area coverage at 20%. This change from the data threshold of 10 co-located pixels in Plant et al., 2022 to a 20% area coverage (which corresponds to more than 10 pixels) impacts the amount of data we have and removes some cities from analysis completely. One could choose different thresholds depending on tolerance for low data days, and have more or less possible observing days.

Table S3. Filtering schemes applied to TROPOMI CH_4 and CO data adopted from Plant et al., 2022

TROPOMI Variable	Species	Filter
scattering_optical_thickness_SWIR	CO	< 0.5
height_scattering_layer	CO	< 5000
carbonmonoxide_total_column_precision (converted to dry column precision)	CO	$<= 5$
surface_albedo_2325	CO	$>= 0.02$
aerosol_optical_thickness_SWIR<br>or methane_mixing_ratio_precision	CH_4	< 0.07<br>$<= 10$
surface_albedo_SWIR	CH_4	$>= 0.02$ (Hu. et al 2016 AMT)
qa_value	CO	> 0
qa_value	CH_4	> 0
surface_classification	CO, CH_4	%% 2 (not over water)

Table S4. Filtering schemes applied during enhancement ratio analysis

Species	Filter	Source
CO, CH_4	$>= 20\%$ area covered by co-located pixels	this work
CO	$>= 7$ ppb/pixel	Plant et al., 2022
CO	cumulative fire emissions < 57 MgCO/h within 1.5° from city center<br>burn events < 23 MgCO/h within 0.75° from city center	Leguijt et al, 2023

Table S5. Data availability for the cities as of 2023 after filtering, sorted by C40-designated regions followed by non-C40 cities. The average number of observations is the mean of the annual average observations of every city with data across all years (2019-2023). The average relative 95%CI represents regional mean of the 95% confidence interval of every city with data across all years (2019-2023).

C40 Region	# C40 Cities	# Cities with at least 1 Year of Data	# Cities with Data 2019 - 2023	Average number of observations per city per year	Average relative 95%CI for detection of change
Africa	11	6	2	36.3	25.6%
Central East Asia	11	9	7	34.9	27.4%
East, Southeast Asia & Oceania	12	6	6	21.1	31.4%
Europe	17	16	12	16.1	35.5%
Latin America	13	8	5	30.8	26.9%
North America	17	16	11	22.4	30.4%
South & West Asia	10	10	8	45.4	27.0%
Non-C40 cities		21	21	26.3	30.1%

The amount of data that passes filtering has increased across the globe as years pass. We explore whether this could be causing the increasing emissions that we observe in Figure S4, but this seems unlikely as the regional relationships to data

availability do not match the overall increases in enhancement ratio and emissions seen in almost every region except Europe.

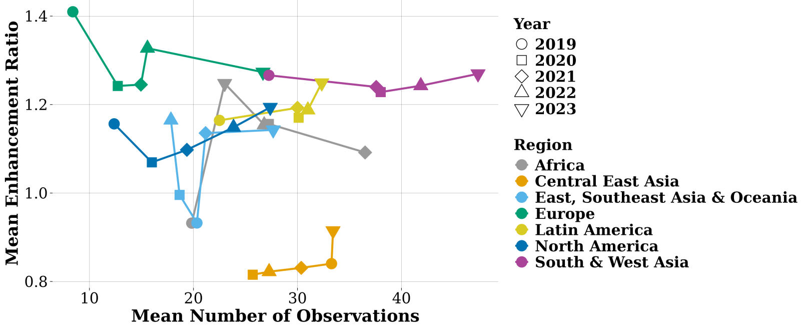

Fig. S4. Number of observations in a city per year averaged by region compared to the annual average enhancement ratio for the same region. The enhancement ratios are increasing in time in most regions and the average number of observations increases in most regions, but the increase in both does not seem to be linked as regions show differing relationships. The increase in observations that meet filtering requirements per year does not appear to drive observations of increasing urban CH_4 emissions.

Additionally, we consider how TROPOMI measurements may be impacted by correlation of the column measurements with aerosol optical thickness (AOT) and surface albedo. Figure S5 shows the percent change in the annual enhancement ratio averaged by region. Noticeably, there is a larger negative correlation of CH_4 AOT with the CH_4 mixing ratio, but this is also where we see wider confidence intervals in the enhancement ratios, likely smoothing any sensitivity of our analysis to this small correlation.

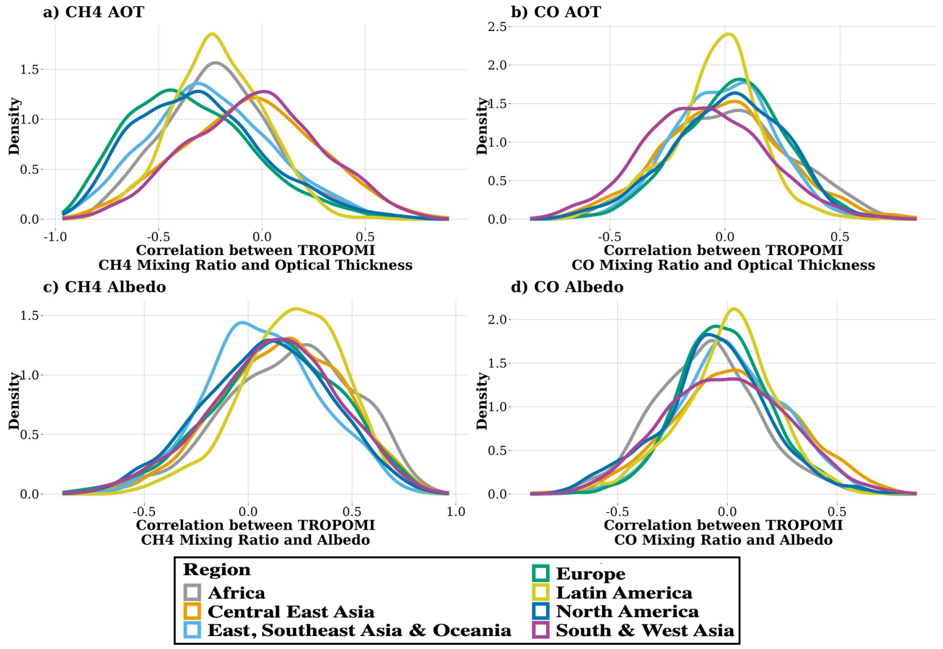

Fig. S5. Kernel density estimation of correlation between a) TROPOMI CH_4 mixing ratio and aerosol optical thickness; b) TROPOMI CO mixing ratio across and aerosol optical thickness; c) TROPOMI CH_4 mixing ratio and CH_4 albedo; and d) TROPOMI CO mixing ratio and CO albedo for each daily TROPOMI overpass, grouped by region.

4. Uncertainty on the Annual Enhancement Ratio and Annual Emissions

A. Uncertainty Methodology. First, we quantify uncertainty in calculating annual enhancement ratios, accounting for instrumental, retrieval, methodological, and data-driven variability. For each satellite overpass, we calculate the enhancement ratio for a variety of background percentiles (10%-20%). To propagate uncertainty in the daily enhancement ratios for each background choice, we apply the mean retrieval precisions of the TROPOMI CH_4 and CO column mixing ratios as the standard deviations. Following Plant et al., 2022, the CH_4 retrieval precision is doubled to better align with ground-based validation efforts (16, 27). We resample and average the daily enhancement ratios 1000 times, generating a distribution of annual mean enhancement ratios for each city and year. Finally, we apply bootstrapping to the annual enhancement ratio realizations to estimate the annual mean and associated confidence intervals. We evaluate the impact of sampling the background percentile between 10% and 20% separately for CH_4 and CO and find the annual enhancement ratios are not sensitive to the percentile choice

differing between the two enhancements, shown in Figure S6. We also evaluate keeping the background percentile constant within the annual resampling rather than randomly sampling, shown in Figure S7. We find the annual enhancement ratios are robust to systematic error that could emerge from consistent background percentile selection. The methodology for quantifying uncertainty in the annual enhancement ratios is illustrated in Figure S9(a).

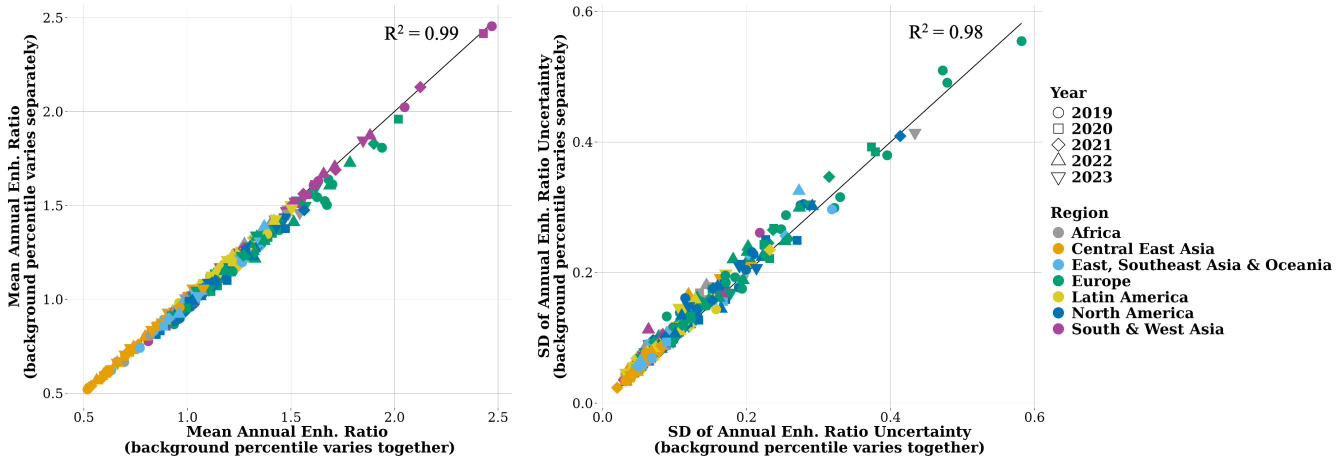

Fig. S6. Mean (left) and standard deviation (right) of the error distributions of city annual enhancement ratios. The x-axis shows the annual enhancement ratio for each city if the enhancements for both gases are found by subtracting off a background using the same percentile value between 10% and 20%, whereas the y-axis shows the annual enhancement ratio if the enhancements are found by subtracting off different background values between 10% and 20% for each gas. In both cases, the background percentiles vary in each iteration. The year of the annual enhancement ratio is denoted by the shape of the point and the city region is denoted by the point color. The 1:1 line is shown in black and the R-squared value is located in the top right of each panel.

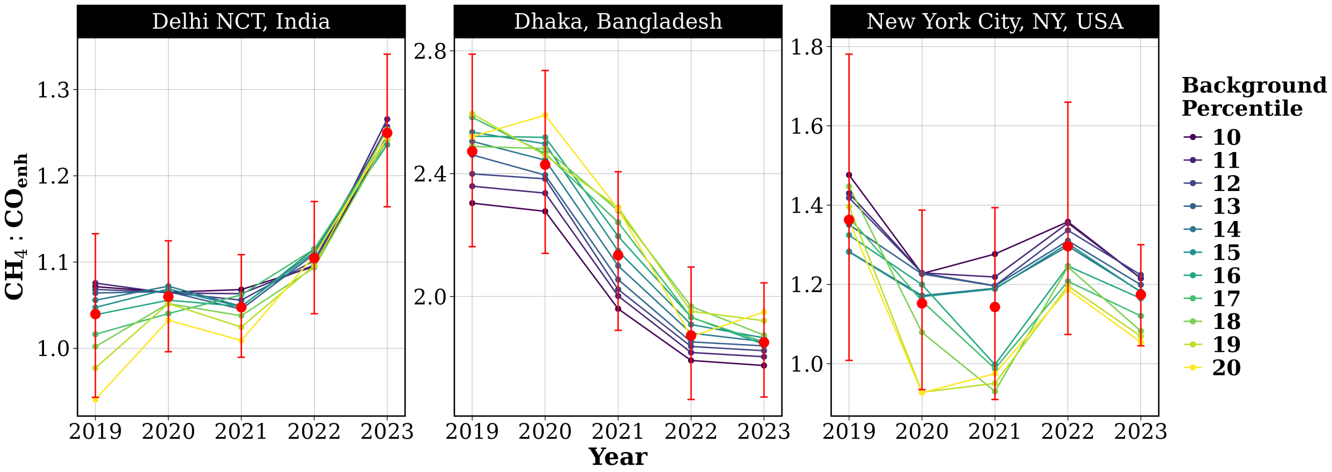

Fig. S7. The annual enhancement ratios for Delhi, Dhaka and New York City are shown by the lines ranging from purple to yellow, depending on the background percentile used to isolate the enhancements. We additionally plot the annual enhancement ratios, where the background percentile is randomly selected from the 10-20th percentile as used in this work and 95% confidence intervals in red. The background percentile-specific enhancement ratios are within the confidence intervals of the annual enhancement ratios.

Then, to calculate the absolute uncertainty inon the annual CH_4 emissions for a city, we combine the 1000 numerical realizations of the annual enhancement ratio with numerical realizations of the EDGAR v8.1 CO gridded inventory emissions total. To calculate the absolute uncertainty in the EDGAR v8.1 CO emissions, we sum the EDGAR v8.1 CO annual emissions over the grid cells within the urban domain. Then we use the regional uncertainties listed in the Supporting Information of Crippa et al., 2018 (28) to apply Gaussian noise to the CO total for a city. The resulting 1000 numerical realizations of the EDGAR v8.1 CO emissions incorporates the absolute uncertainty in CO emissions for a specific city and year. Using the uncertainty provided in Crippa et al., 2018 is conservative when compared to other methods used in similar approaches (25).

Separately, to calculate confidence in how the CH_4 emissions evolve or change over time, we additionally consider possible changes in CO emissions that may not be included in the direct conversion from enhancement ratio to CH4 emissions. Known changes in CO emissions, as reported in the EDGAR inventory, are explicitly included in the analysis. In cases with rapid CO emission change reported in the inventory, the resultant trajectory of estimated CH4 emissions are not driven by the inventoried CO change, as seen in two examples discussed in SI Section 5. To account for the possibility of additional changes in CO

emissions, we introduce Gaussian noise (σ=12%/y) to the CO inventory total for a city that is an observationally-informed, but conservative representation of how CO emissions could change over time. This value is based on an observational cross sectional flux (CSF) study using the methodology of Leguijt et al., 2023 (29), which used TROPOMI to quantify daily and annual CO emissions from 67 urban cores from 2019 to 2021. We calculated the geometric mean annual percent change between 2019 and 2021 CO emissions across all 67 urban cores to be -12%. Of the cities observed, 43 cities are included in both studies. The urban area described by the CSF emissions are, on average, 7% of the urban domain area used in this work. Applying this observed decline to the entire urban domain would be inconsistent with how global trends in anthropogenic CO emissions reported in Zheng et al., 2019 (30). Therefore, we use the observed variability over urban cores to impose noise without assuming a direction, conservatively accounting for additional changes in CO emissions over time. We also evaluated replacing the urban core CO emissions in EDGAR v8.1 with the annual CO emissions observed in the CSF study. We show the annual CO emissions with the replaced core compared to the original EDGAR v8.1 CO emissions for the 43 cities in Figure S8(a). In Figure S8(b), we show the annual CH_4 emissions quantified when using the CO emission data from EDGAR with the urban core area replaced by the CSF CO observations. Overall, incorporating observationally derived CO emissions into calculation of urban CH_4 emissions leads to an increase in observed CH_4 emissions in a majority of the 43 cities but limits the number of cities and years available for evaluation.

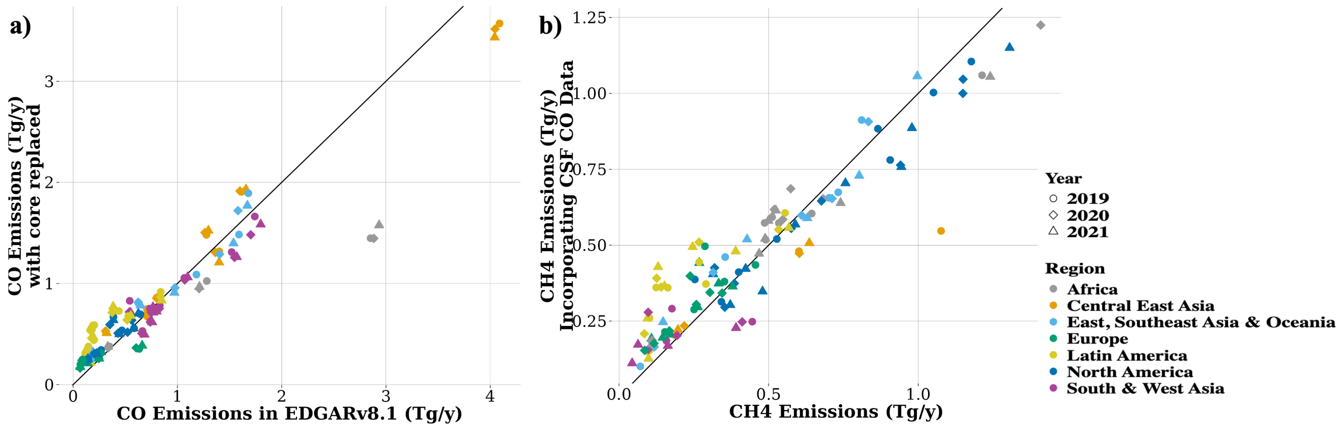

Fig. S8. (a) Annual urban EDGAR v8.1 CO emissions for our urban domain compared to those where the urban core emissions are replaced by the observational CSF study result. (b) Annual CH_4 emissions observed for each city in this study using the CO emission data from EDGAR with the urban core area replaced by the CSF CO observations. The black lines in (a) and (b) show a one-to-one line. Incorporating the CSF study results into the CH_4 observations leads to an increase in estimated CH_4 emissions in cities evaluated in both studies across Europe; Latin America; South & West Asia; and Central East Asia. The CSF CO emissions suggest EDGAR v8.1 overestimates CO emissions in some cities in East, Southeast Asia & Oceania and North America.

We illustrate both calculations of uncertainty in the annual CH_4 emissions in Figure S9(b). In both cases, the "noisy" annual enhancement ratios are multiplied element-wise by the "noisy" CO emissions and by the scalar ratio of CH_4 and CO molecular masses. We apply bootstrapping to the resulting numerical realizations of the annual CH_4 emissions for a given city for a given year to estimate the mean and associated 95% confidence intervals to represent the city's annual observed CH_4 emissions. Additionally, we can aggregate the distribution of annual CH_4 emissions with that of other cities to find regionally summed CH_4 emissions, for example.

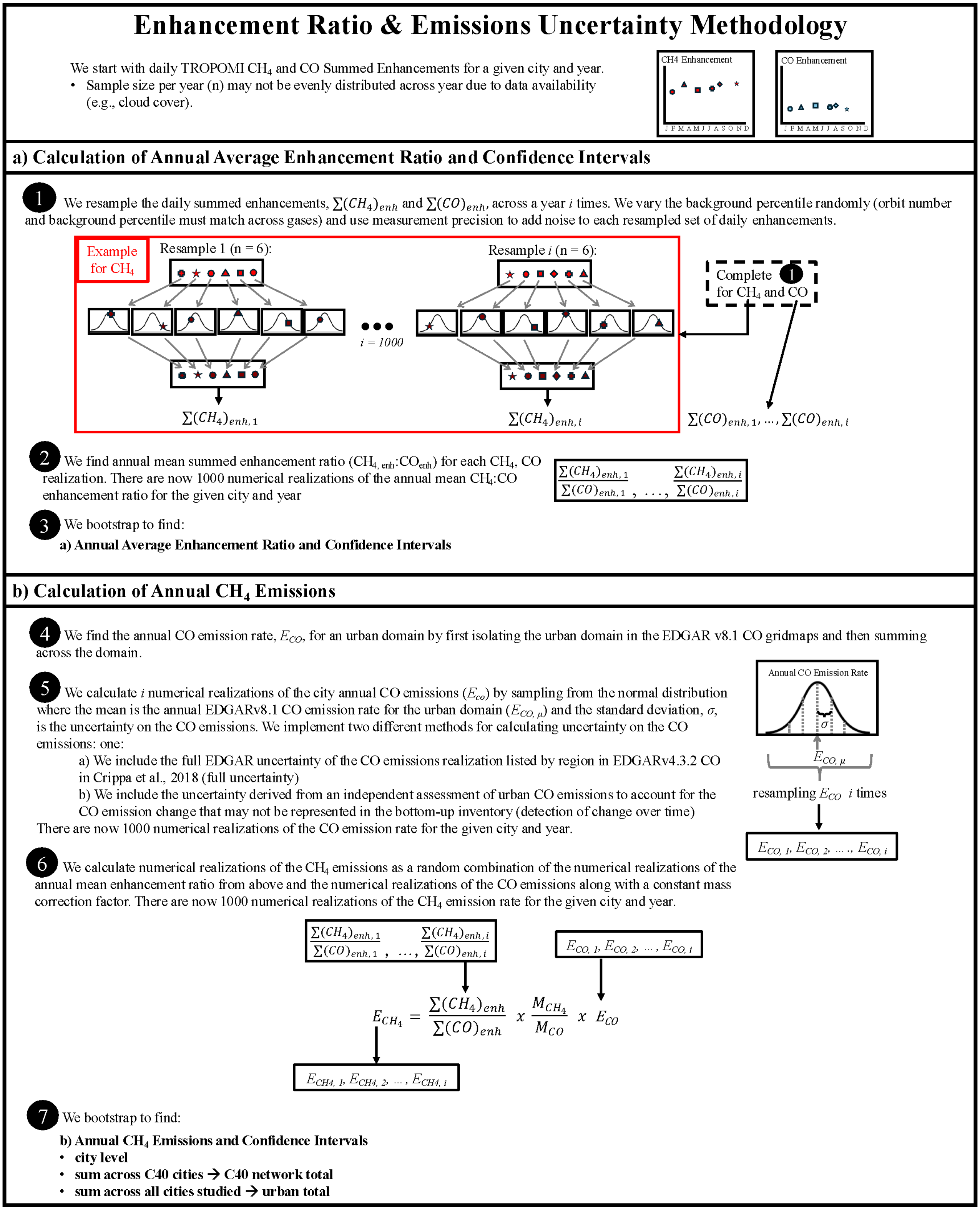

Fig. S9. Flow chart showing each step of calculating uncertainty of the annual enhancement ratio (a) and annual CH_4 emissions (b).

B. Interpretation of Change. We evaluate how CH_4 emissions change by first formulating distributions for the annual CH_4 emissions that incorporate uncertainty from the CSF study discussed previously on the annual CO emission value with uncertainty on the annual enhancement ratios, following Eqn. 4 as shown in the Methods. We can aggregate (e.g. regionally) to evaluate how the total emissions of specific groups of cities behave over time. The distributions of the realizations for the summed annual emissions for C40 and non-C40 cities with the uncertainty from the detection of change are shown for each year from 2019 through 2023 in Figure S10. We calculate the annual mean emissions and 95% confidence intervals for both groups across years (see Table S2).

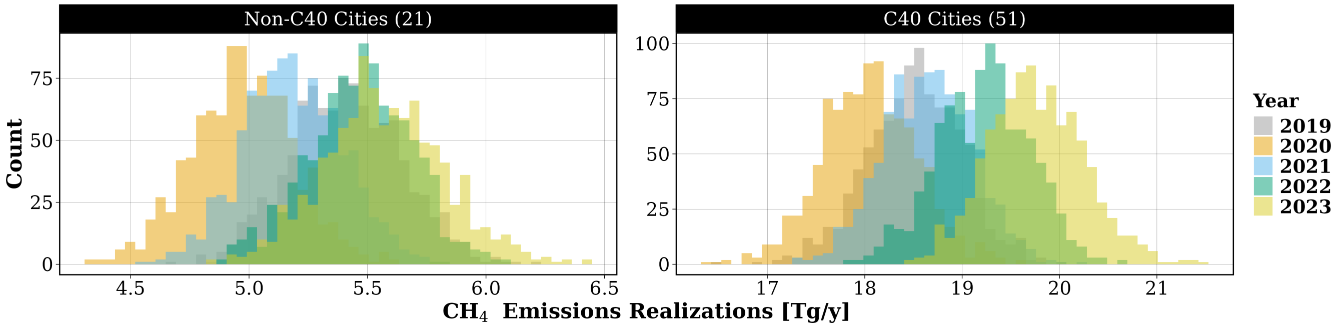

Fig. S10. Distributions of the summed annual CH_4 emissions of aggregated C40 (right) and non-C40 cities (left). The number of cities that went into each annual sum is noted in parenthesis in each panel title. The color of the histogram denotes the year from 2019 through 2023. The distributions are each composed of 1000 realizations of the aggregated CH_4 emission sum, as described in SI S4: Uncertainty on the Annual Enhancement Ratio and Annual Emissions.

In the utilized uncertainty approach, the number of annual emissions realizations is large such that formal statistical testing (e.g., t-test) is not appropriate. To meaningfully speak to the detection of difference between years, we calculate a difference distribution by sampling 5000 times in a Monte-Carlo approach from the two annual groups and finding the difference between the values each time. The difference distributions are shown in Figure S10 for different groupings of years for the C40 city network and for non-C40 cities. We report the mean of the difference distribution as the change in emissions between the two years. We find confidence intervals for the difference distributions at the 90 and 95% confidence level (90%CI and 95%CI, respectively). If the confidence interval includes zero, there is no significant difference between the years at that confidence level. In some cases, there is significance at the 90% confidence level but not at the 95% confidence level. We report both confidence interval values, 95%CI and 90%CI, in the manuscript, where significance is found at the confidence levels that do not overlap 0. Figure S10 indicates that the difference distribution for both sets of cities from 2019 to 2020 in dark blue is distinct from the difference distributions for other individual steps in years. However, this distinction does not meet the threshold for statistical significance. For the C40 cities, a statistically detectable change is observed from 2019 to 2023 at the 90%CI and 2020 to 2023 at both the 95%CI and 90%CI, while statistically detectable change is observed from non-C40 cities from 2020 to 2023 and is significant only at the 90%CI.

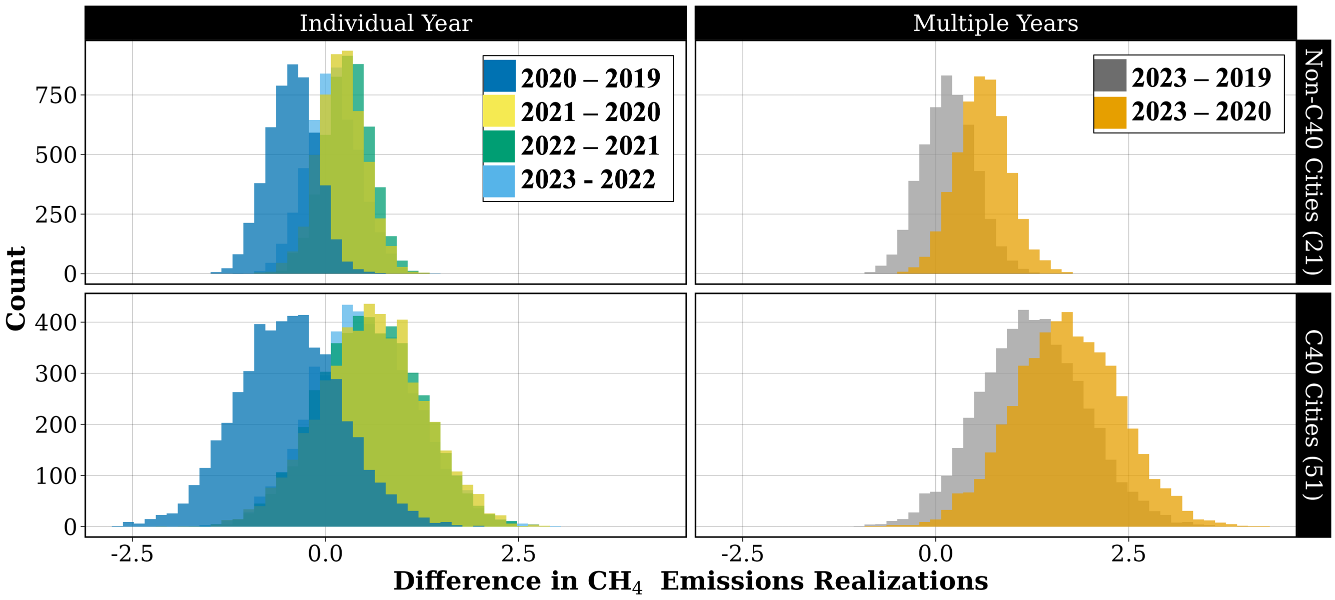

Fig. S11. Difference distributions of CH_4 emissions for specific pairs for years for non-C40 cities (top row) and C40 cities (bottom row). The number of cities that went into group is noted in parenthesis in each row title. Each difference distribution is composed of 5000 realizations of the aggregated CH_4 found in a Monte-Carlo approach for the specified group of cities. The changes between individual years (ex: 2019 to 2020) are in the left panels whereas the changes between multiple years (ex: 2019 to 2023) are in the right panels. Difference distributions across multiple years directly compare the years listed only.

5. CO Inventory

A. EDGAR CO v8.1. The EDGAR v8.1 CO gridmaps used in this study were downloaded after August 2024. Initially, we observed a discrepancy between annual CO emissions rates if the CO data was calculated using the monthly gridmaps (downloaded after August 2024) or the annual gridmaps (downloaded before August 2024). At the global scale, this discrepancy is small, the annual total CO emission is 12 tonnes lower than annual total CO emissions calculated with monthly emissions data. The discrepancy widens at the city level; for example, the annual total CO emissions for Washington, DC are 10% lower when using annual CO gridmap files downloaded earlier compared to using monthly CO gridmap files downloaded later. The CO emission rates in the annual gridmaps changed between April and August 2024 although version and file names remained the same. We cannot confirm if the monthly CO files also changed at this time as we only had newer monthly files. The discrepancy between monthly and annual files disappears if files downloaded only later than August are used.

B. Case Studies of Large EDGAR CO v8.1 Change: Kyiv and Istanbul. Annual EDGAR v8.1 CO emissions in 2023 are on average only 0.46% lower than 2019 over the urban domains of this study. Where the CO inventory does predict larger changes, the atmospheric CO changes commensurately leading to very small changes in CH_4 emissions. For example, the inventory shows the strongest decline in inventoried CO emissions in Kyiv, Ukraine with emissions in 2023 reported as 18.1% smaller than 2019. If the changing CO emissions inventory drove the CH_4 emissions, we would observe declining CH_4 emissions at a similar rate. However, we observe the annual enhancement ratio of Kyiv to increase from 1.08 to 1.55 and the annual CH_4 emissions to increase incrementally from 0.08 to 0.09 Tg/y from 2019 to 2023, demonstrating that changes in inventoried CO emissions do not cause an interpretation of the same change in observed CH_4 emissions. Istanbul in Türkiye shows the strongest increase in inventoried CO emissions with 2023 emissions being 20% larger than 2019. We observe the annual enhancement ratio to decrease from 1.66 to 1.44 and resulting in observed CH_4 emissions to change from 0.23 to 0.24 Tg/y. On top of accounting for inventoried changes in CO emissions, we incorporate changes in CO emissions not reflected in the inventories estimates. The annual observed CH_4 emissions changes from 0.08 to 0.09 Tg/y in Kyiv and 0.23 to 0.24 Tg/y in Istanbul are within our confidence intervals for the detection of change. Therefore, we do not detect significant change in CH_4 emissions in this city even though CO emissions changed largely over the same time.

C. Shanghai, China. The EDGAR v8.1 CO Inventory provides reported, globally gridded CO emissions until 2022. Our study period is 2019 through 2023 so we use the 2022 emissions value for both 2022 and 2023. The CO Emissions estimated across the past 3 versions of EDGAR for Shanghai, China are upwards of 250 kg/s, which is significantly larger than other cities. For this city, we scale the EDGAR v8.1 CO emissions down with the Hemisperic Transport of Air Pollution mosaic (HTAPv3), a mosaic inventory at the same native resolution as EDGAR that pulls from the Regional Emission inventory in ASia (REAS) for most of the Asian domain (31). The latest year of available HTAPv3 data is 2018, so this year of data is used to scale the EDGAR CO Inventory data from 2019-2023 for Shanghai, China. EDGAR v8.1 CO is used as the CO inventory for every city except Shanghai in order to create a consistent method.

D. Other CO Inventories. This study does not compare the use of other inventories in place of EDGAR v8.1 CO because there are no other current global gridded inventories. We compare using EDGAR v8.1 CO with using the 2018 CO emissions from HTAPv3 to scale the EDGAR v8.1 CO data from 2019-2023. Figure S12 shows the observed CH_4 emissions of cities when HTAPv3 is used to scale EDGAR v8.1 CO emissions compared to using EDGAR v8.1 CO unscaled in the tracer-tracer approach. The changes seen in the emissions estimates follow a strict regional pattern because HTAPv3 is a regional mosaic inventory. The changes in the observed estimates do not include temporal changes as we used one year (2018, the last year of data) to scale the EDGAR data. The emissions sources that are compiled into HTAPv3 include EMEP for Europe (CAMS-REG-v5.1), the US Environmental Protection Agency over the US, Environment and Climate Change Canada (ECCC) over Canada, REAS for most of the Asian domain, JAPAN for Japan, and CAPPS-KU for Korea (31). For countries not mentioned, HTAPv3 pulls gridded CO emissions from EDGAR v6.1, which was last updated in 2018 (32). For regions where EDGAR v6.1 is used to scale the EDGAR v8.1 data, such as Africa; Latin America; and some of East, Southeast Asia & Oceania, the observed CH_4 emissions are less than originally calculated with EDGAR v8.1. However, in regions where a local inventory is used, the observed CH_4 emissions are higher. We use EDGAR v8.1 CO in this work to keep globally consistent and most up-to-date. Additionally, Mastrogiacomo et al., 2025 concludes EDGAR v8.1 CO provides the best bottom-up representation of CO emissions over global urban regions.

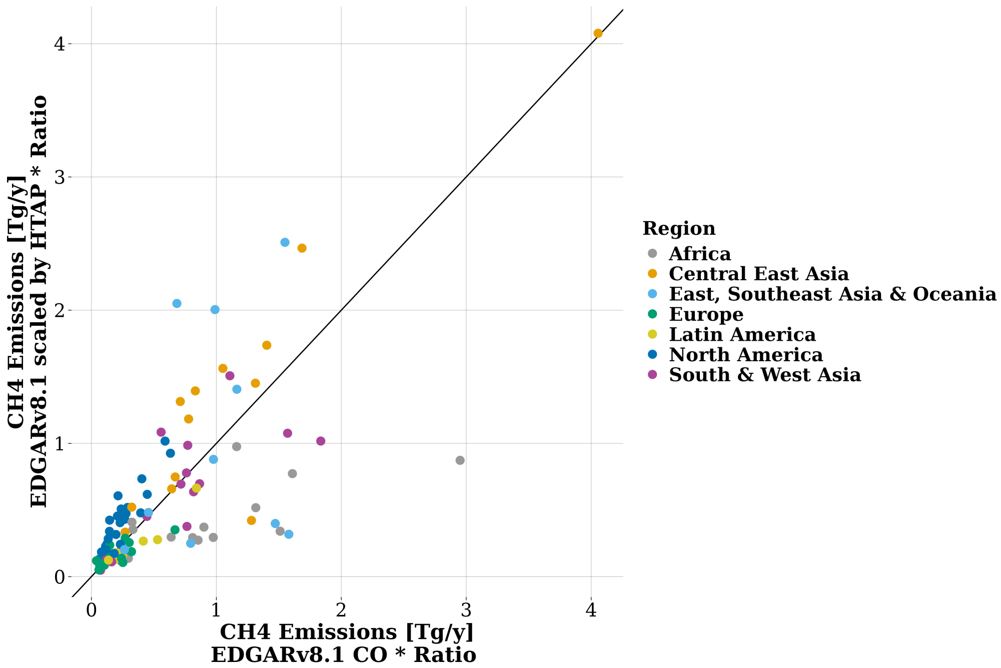

Fig. S12. Annual observed CH_4 emissions in Tg/y with the EDGAR v8.1 CO inventory data in 2023 is scaled by the 2018 HTAPv3 sum for the urban domain before multiplying by a mass correction term and enhancement ratio as shown in Methods compared to the CO emissions inventory being unscaled. CH_4 Emissions reported throughout this work utilize the unscaled EDGAR v8.1 CO gridded emissions as the inventory in tracer-tracer approach.

6. Seasonal Emissions

A. Seasonal CH_4 Emissions. Seasonal urban CH_4 emissions have been quantified in a handful of cities studied here, such as Washington, DC; Baltimore; Boston; Los Angeles, and London (5, 8, 9, 11, 13, 15, 26, 33–37). Often, a seasonal relationship emerges between natural gas consumption and CH_4 emissions. It is not clear, however, if seasonal variability observed in these studies is representative of other cities and regions. Our current global analysis is at an annual scale. However, our annual CH_4 estimates for cities with previously characterized seasonality are comparable. For example, Sargent et al., 2021 finds modest seasonality in Boston with average CH_4 emissions from 2012 to 2020 of 0.20 ± 0.05 Tg/y and Mueller et al., 2025 reports annual CH_4 emissions from 2017 to 2020 to be 0.15 ± 0.03 Tg/y, with no significant difference between seasons. Consistently, we find annual average CH_4 emissions from 2019 to 2020 to be 0.15 Tg/y in Boston. In Los Angeles, Wong et al., 2016 derives annual CH_4 emissions across 2011-2015 to be 0.34 Tg/y; Yadav et al., 2019 finds 0.33 ± 0.09 Tg/y for 2015-2016; He et al., 2019 finds 0.41 Tg/y for 2011-2017; and Zeng et al., 2023 finds 0.42 Tg/y for 2011-2021. These studies suggest winter CH_4 emissions are 40% higher than summer emissions, however some studies report a secondary summertime emission peak in addition to the maximum in winter (e.g. Wong et al., 2016). We calculate the average CH_4 emissions of Los Angeles from 2019 to 2020 to be 0.27 Tg/y which is comparable with Yadav et al., 2019, but is generally smaller than previous studies. However, our urban domain for Los Angeles is smaller than previous studies and does not include much of counties east of Los Angeles county that are included in previous studies. In the Washington, DC and Baltimore metropolitan area, Huang et al., 2019 estimates

annual CH_4 emissions to be 0.47 ± 0.1 Tg/y in 2016; Karion et al., 2023, states that annual emissions averaged over 2018 to 2021 are 0.13 ± 0.03 Tg/y; and Mueller et al., 2025 finds annual emissions averaged over 2017 through 2021 to be 0.31 ± 0.08 Tg/y. We calculate emissions to be 0.23 Tg/y from 2019-2021. Karion et al., 2023; Huang et al., 2019; and Mueller et al., 2025 all find moderate seasonality with wintertime CH_4 emissions being 44%, 41%, and 24% higher, respectively. Outside of the United States, Helfter et al., 2016 finds mean annual emissions to be 0.12 Tg/y over a 2011-2012 period, with 21% larger CH_4 emissions in winter than in summer. Across our study period, we find average emissions to be 0.12 Tg/y for London as well.

B. Seasonal CO Emissions. While TROPOMI passes over each city once per day, meteorology, especially monsoons or cloudy seasons, strongly impacts how much data passes filtering requirements and is available for analysis. We consider how seasonal gaps as seen in Figure 1(f)-(g) may impact how our data represents an annual emissions estimate. We find that our data availability after filtering is often consistent over time. While we have more data meeting filtering thresholds in later years, these overpasses more often occur in months for which we have data in previous years. The time of year which our data covers is consistent. This is important because EDGAR v8.1 CO split by month does not show significant, if any, change in emissions patterns for the same month from year to year (38). EDGAR v8.1 CO suggests the CO emissions fluctuate throughout the year in different regions, but as the number of annual observations increases across time, it often increases in months where data meets filtering requirements in previous years. In this work, the annual CO emission rate for each city is used with the annual enhancement ratio to calculate the annual CH_4 emissions. For the two cities shown in Figure 1(f)-(g), Figure S13 shows the CH_4 emissions where we instead calculate a mean CO emission rate for the city from EDGAR v8.1 CO monthly gridded emissions for months in which we have observations. These two case studies show that in comparison to the CH_4 emissions calculated in this work, the CH_4 emissions where monthly data was incorporated is within the original 95% confidence intervals and that generally, the trajectory over time is maintained.

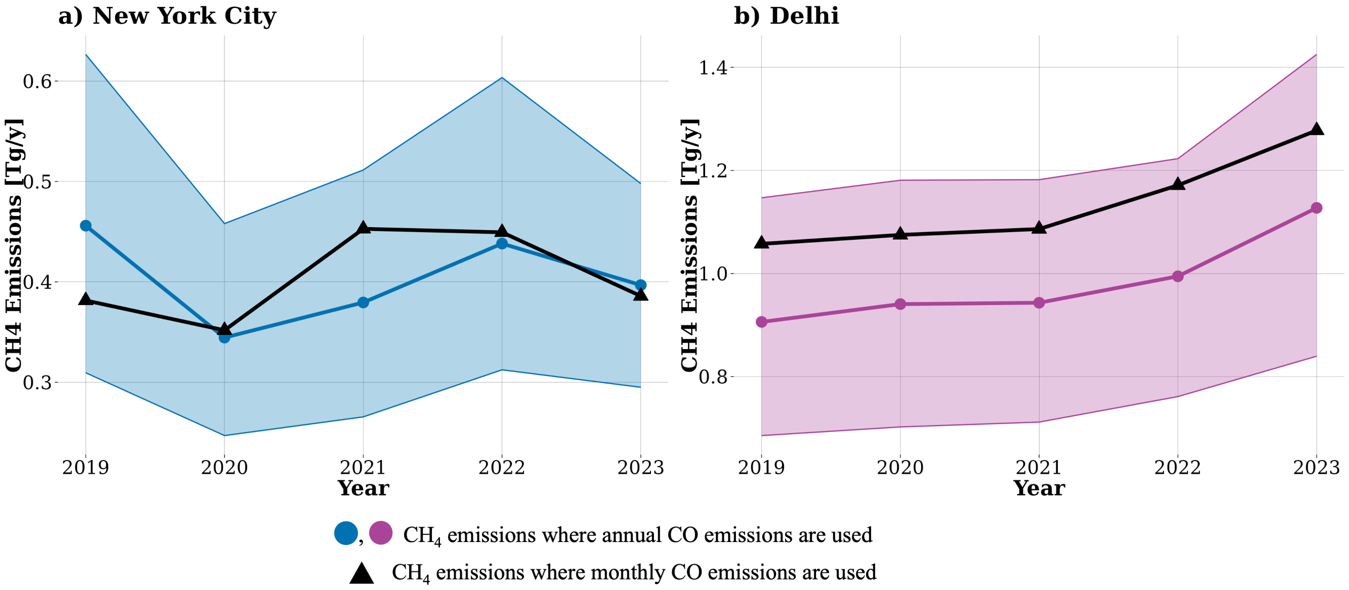

Fig. S13. Annual observed CH_4 emissions in Tg/y for (a) New York City and (b) Delhi compared with CH_4 emissions if an average of monthly CO emissions for months in which we have observations is used in the tracer-tracer approach. The shaded area shows the 95% confidence interval for the annual observed CH_4 emissions. The CH_4 emissions where annual CO emissions are used and the corresponding CI are the same values as reported in Table S1.

7. Comparing City Emissions Across Methods

Past urban methane studies of individual urban regions have reported underestimation of CH_4 emissions, at times by twofold or more, in the Emissions Database for Global Atmospheric Research (EDGAR) bottom-up inventory (7, 10, 13, 14, 18, 19). In comparison with current and previous versions of EDGAR, we do not see such a large underestimation of urban CH_4 emissions. This, however, could be explained by differences in the urban domains used, with smaller domain definitions having larger mismatch in atmosphere - inventory comparisons, as has been established in urban carbon dioxide studies (39). We hypothesize that our large spatial domain capturing the city as a whole reveals a spatial disaggregation error of EDGAR, where city emissions are flattened over surrounding grid cells whereas previous studies compare emissions focused upon the urban core.

There does not exist a uniform domain definition for metropolitan studies, rendering direct comparison inaccurate. Different methodologies require specific domain boundaries to be set to robustly capture urban emissions. Along with urban CH_4 studies quantifying emissions for varying spatial domains, studies occur over different years, times of year, compare to different bottom-up inventories and different versions of these inventories. Spatial differences, due to the lack of a consistent urban boundary and temporal differences across methodologies, lead to apparent discrepancies that make direct comparison of

methodologies ill-posed. In some cases, work has been done to adjust domain sizes to enable comparison, reducing disparities between works, but it is not always feasible (33). Additionally, some urban studies report conclusions in terms of their observation results relative to EDGAR, complicating meaningful comparison across studies as EDGAR updates values change with every EDGAR version. We include comparison to annual emissions from studies that report on urban CH_4 seasonality in SI S6 to evaluate the potential seasonal bias in observation availability, but cannot speak definitively on differences in results due to the differences in spatial extents and periods of study.

8. Investigating Change in Emissions in 2020

There is a slight dip in observed CH_4 emissions in 2020. The global COVID-19 pandemic occurred in 2020 (40) and lead to significant changes in human behavior as many cities enacted stay-at-home or lockdown orders in March through June of 2020 (41–46), except in China where lock-downs started over one month earlier (47). We might expect reductions in CO emissions from reduced on-road vehicles, which would typically cause the $CH_{4,\ enh}$:CO_{enh} ratio to increase and therefore observed CH_4 emissions to increase. In 2020, however, both the ratio and methane emissions decreased. It is important to note CO enhancement and emission reductions were most pronounced during the few months of extensive lock-downs (45). Unfortunately, we have very few satellite-based observations that meet filtering requirements from cities during that critical time. According to EDGAR v8.1, annual total emissions showed small decreases, often amounting to just a few hundredths of a teragram per year. The annual enhancement ratio here is significantly influenced by changes in the CH_4 enhancement, as illustrated by a strong correlation ($r = 0.84$), whereas changes in the CO enhancement are smaller in magnitude and less variable ($r = -0.33$). Theoretically, a lack of observations during lockdowns allows for the annual CO emission rate, which could have some impact to emissions, to inappropriately bolster CH_4 emissions as the time over which impacts to CO emissions occurred were not observed. However, we confirmed that if an adjusted annual CO total for each city was calculated using the only months in which we capture observations, the results of this work are within the confidence intervals. This is also seen in closer depth for New York City and Delhi in Figure S13. As the ratio is driven by change in the CH_4 enhancements and the CO emissions inventory change is very slight if any at all, there is a resultant observed decrease in CH_4 emissions at the annual level. This analysis focuses on the annual scale, allowing the inclusion of a large number of cities for study. This approach is dictated by data availability driven by varying meteorological conditions and satellite sampling. While finer temporal analyses are possible in certain locations, they are not feasible at this global scale.

References

1. C40 Cities Climate Leadership Group, GHG Interactive Dashboard Data (2024).
2. D Wunch, PO Wennberg, GC Toon, G Keppel-Aleks, YG Yavin, Emissions of greenhouse gases from a North American megacity. *Geophys. Res. Lett.* **36** (2009).
3. PO Wennberg, et al., On the Sources of Methane to the Los Angeles Atmosphere. *Environ. Sci. & Technol.* **46**, 9282–9289 (2012).
4. J Peischl, et al., Quantifying sources of methane using light alkanes in the Los Angeles basin, California. *J. Geophys. Res. Atmospheres* **118**, 4974–4990 (2013).
5. K McKain, et al., Methane emissions from natural gas infrastructure and use in the urban region of Boston, Massachusetts. *Proc. Natl. Acad. Sci.* **112**, 1941–1946 (2015).
6. YY Cui, et al., Top-down estimate of methane emissions in California using a mesoscale inverse modeling technique: The South Coast Air Basin. *J. Geophys. Res. Atmospheres* **120**, 6698–6711 (2015).
7. BK Lamb, et al., Direct and Indirect Measurements and Modeling of Methane Emissions in Indianapolis, Indiana. *Environ. Sci. & Technol.* **50**, 8910–8917 (2016).
8. C Helfter, et al., Spatial and temporal variability of urban fluxes of methane, carbon monoxide and carbon dioxide above London, UK. *Atmospheric Chem. Phys.* **16**, 10543–10557 (2016).
9. CK Wong, et al., Monthly trends of methane emissions in Los Angeles from 2011 to 2015 inferred by CLARS-FTS observations. *Atmospheric Chem. Phys.* **16**, 13121–13130 (2016).
10. X Ren, et al., Methane Emissions From the Baltimore-Washington Area Based on Airborne Observations: Comparison to Emissions Inventories. *J. Geophys. Res. Atmospheres* **123**, 8869–8882 (2018).
11. L He, et al., Atmospheric Methane Emissions Correlate With Natural Gas Consumption From Residential and Commercial Sectors in Los Angeles. *Geophys. Res. Lett.* **46**, 8563–8571 (2019).
12. G Plant, et al., Large Fugitive Methane Emissions From Urban Centers Along the U.S. East Coast. *Geophys. Res. Lett.* **46**, 8500–8507 (2019).
13. Y Huang, et al., Seasonally Resolved Excess Urban Methane Emissions from the Baltimore/Washington, DC Metropolitan Region. *Environ. Sci. & Technol.* **53**, 11285–11293 (2019).
14. NV Balashov, et al., Background heterogeneity and other uncertainties in estimating urban methane flux: results from the Indianapolis Flux Experiment (INFLUX). *Atmospheric Chem. Phys.* **20**, 4545–4559 (2020).
15. MR Sargent, et al., Majority of US urban natural gas emissions unaccounted for in inventories. *Proc. Natl. Acad. Sci.* **118** (2021).
16. G Plant, EA Kort, LT Murray, JD Maasakkers, I Aben, Evaluating urban methane emissions from space using TROPOMI methane and carbon monoxide observations. *Remote. Sens. Environ.* **268**, 112756 (2022).

17. JR Pitt, et al., New York City greenhouse gas emissions estimated with inverse modeling of aircraft measurements. *Elem. Sci. Anthropocene* **10** (2022).
18. JD Maasakkers, et al., Using satellites to uncover large methane emissions from landfills. *Sci. Adv.* **8** (2022).
19. B de Foy, JJ Schauer, A Lorente, T Borsdorff, Investigating high methane emissions from urban areas detected by TROPOMI and their association with untreated wastewater. *Environ. Res. Lett.* **18**, 044004 (2023).
20. Z Xing, TE Barchyn, C Vollrath, M Gao, C Hugenholtz, Satellite-Derived Estimate of City-Level Methane Emissions from Calgary, Alberta, Canada. *Remote. Sens.* **16** (2024).
21. M Hemati, M Mahdianpari, R Nassar, H Shiri, F Mohammadimanesh, Urban methane emission monitoring across North America using TROPOMI data: an analytical inversion approach. *Sci. Reports* **14** (2024).
22. H Nesser, et al., High-resolution US methane emissions inferred from an inversion of 2019 TROPOMI satellite data: contributions from individual states, urban areas, and landfills. *Atmospheric Chem. Phys.* **24**, 5069–5091 (2024).
23. JR Pitt, et al., Underestimation of Thermogenic Methane Emissions in New York City. *Environ. Sci. & Technol.* **58**, 9147–9157 (2024).
24. H Ohyama, Y Yoshida, T Matsunaga, CH4 and CO emission estimates for megacities: deriving enhancement ratios of CO2, CH4, and CO from GOSAT-2 observations. *Environ. Res. Lett.* **19**, 124025 (2024).
25. J Mastrogiacomo, M Crippa, CG MacDonald, CM Roehl, D Wunch, Estimating Urban CH_4 Emissions From Satellite-Derived Enhancement Ratios of CH_4, CO_2, and CO. *J. Geophys. Res. Atmospheres* **130** (2025).
26. KL Mueller, et al., Scaling Urban Methane Emissions: Utility of Single-Site Measurements in Five Urban Domains. *Environ. Sci. & Technol.* **59**, 14399–14409 (2025).
27. J Landgraf, et al., ATM-MPC Mission Performance Cluster Methane [L2___CH4____] Readme, (European Space Agency), Technical report (2024).
28. M Crippa, et al., Gridded emissions of air pollutants for the period 1970–2012 within EDGAR v4.3.2. *Earth Syst. Sci. Data* **10**, 1987–2013 (2018).
29. G Leguijt, et al., Quantification of carbon monoxide emissions from African cities using TROPOMI. *Atmospheric Chem. Phys.* **23**, 8899–8919 (2023).
30. B Zheng, et al., Global atmospheric carbon monoxide budget 2000–2017 inferred from multi-species atmospheric inversions. *Earth Syst. Sci. Data* **11**, 1411–1436 (2019).
31. M Crippa, et al., The HTAP_v3 emission mosaic: merging regional and global monthly emissions (2000–2018) to support air quality modelling and policies (2023).
32. M Crippa, et al., EDGAR v6.1 global air pollutant emissions (2022).
33. A Karion, et al., Methane Emissions Show Recent Decline but Strong Seasonality in Two US Northeastern Cities. *Environ. Sci. & Technol.* **57**, 19565–19574 (2023).
34. JK Hedelius, et al., Southern California megacity CO2, CH4, and CO flux estimates using ground- and space-based remote sensing and a Lagrangian model. *Atmospheric Chem. Phys.* **18**, 16271–16291 (2018).
35. V Yadav, et al., Spatio-temporally Resolved Methane Fluxes From the Los Angeles Megacity. *J. Geophys. Res. Atmospheres* **124**, 5131–5148 (2019).
36. V Yadav, et al., A declining trend of methane emissions in the Los Angeles basin from 2015 to 2020. *Environ. Res. Lett.* **18**, 034004 (2023).
37. ZC Zeng, et al., Decadal decrease in Los Angeles methane emissions is much smaller than bottom-up estimates. *Nat. Commun.* **14** (2023).
38. M Crippa, et al., EDGAR v8.1 Global Air Pollutant Emissions (2024) [data set].
39. CK Gately, LR Hutyra, Large Uncertainties in Urban-Scale Carbon Emissions. *J. Geophys. Res. Atmospheres* **122** (2017).
40. N Zhu, et al., A Novel Coronavirus from Patients with Pneumonia in China, 2019. *New Engl. J. Medicine* **382**, 727–733 (2020).
41. A Wilder-Smith, DO Freedman, Isolation, quarantine, social distancing and community containment: pivotal role for old-style public health measures in the novel coronavirus (2019-nCoV) outbreak. *J. Travel. Medicine* **27** (2020).
42. G Dantas, B Siciliano, BB França, CM da Silva, G Arbilla, The impact of COVID-19 partial lockdown on the air quality of the city of Rio de Janeiro, Brazil. *Sci. The Total. Environ.* **729**, 139085 (2020).
43. S Mahato, S Pal, KG Ghosh, Effect of lockdown amid COVID-19 pandemic on air quality of the megacity Delhi, India. *Sci. The Total. Environ.* **730**, 139086 (2020).
44. N Haider, et al., Lockdown measures in response to COVID-19 in nine sub-Saharan African countries. *BMJ Glob. Heal.* **5**, e003319 (2020).
45. LWA Chen, LC Chien, Y Li, G Lin, Nonuniform impacts of COVID-19 lockdown on air quality over the United States. *Sci. The Total. Environ.* **745**, 141105 (2020).
46. Centers for Disease Control and Prevention, CDC Museum COVID-19 Timeline (2024).
47. H Lau, et al., The positive impact of lockdown in Wuhan on containing the COVID-19 outbreak in China. *J. Travel. Medicine* **27** (2020).
